# Evaluating Ovarian Cancer Chemotherapy Response Using Gene Expression Data and Machine Learning

**DOI:** 10.3390/biomedinformatics4020077

**Published:** 2024-05-22

**Authors:** Soukaina Amniouel, Keertana Yalamanchili, Sreenidhi Sankararaman, Mohsin Saleet Jafri

**Affiliations:** 1 School of System Biology, George Mason University, Fairfax, VA 22030, USA; 2 School of Engineering, Brown University, Providence, RI 02912, USA; 3 Department of Biomedical Engineering, The John Hopkins University, Baltimore, MD 21218, USA; 4 Center for Biomedical Engineering and Technology, University of Maryland School of Medicine, Baltimore, MD 21201, USA

**Keywords:** ovarian cancer, chemotherapy, machine learning, drug response prediction, gene expression, feature selection

## Abstract

**Background:**

Ovarian cancer (OC) is the most lethal gynecological cancer in the United States. Among the different types of OC, serous ovarian cancer (SOC) stands out as the most prevalent. Transcriptomics techniques generate extensive gene expression data, yet only a few of these genes are relevant to clinical diagnosis.

**Methods:**

Methods for feature selection (FS) address the challenges of high dimensionality in extensive datasets. This study proposes a computational framework that applies FS techniques to identify genes highly associated with platinum-based chemotherapy response on SOC patients. Using SOC datasets from the Gene Expression Omnibus (GEO) database, LASSO and varSelRF FS methods were employed. Machine learning classification algorithms such as random forest (RF) and support vector machine (SVM) were also used to evaluate the performance of the models.

**Results:**

The proposed framework has identified biomarkers panels with 9 and 10 genes that are highly correlated with platinum–paclitaxel and platinum-only response in SOC patients, respectively. The predictive models have been trained using the identified gene signatures and accuracy of above 90% was achieved.

**Conclusions:**

In this study, we propose that applying multiple feature selection methods not only effectively reduces the number of identified biomarkers, enhancing their biological relevance, but also corroborates the efficacy of drug response prediction models in cancer treatment.

## 1. Introduction

Ovarian cancer (OC) is considered to be the most lethal gynecological cancer in the United States [1,2]. At present, there is no effective screening for OC, resulting in a substantial number of cases being diagnosed at advanced stages of cancer characterized by tumor metastasis. Epithelial ovarian cancer (EOC) is the most common type of OC, accounting for more than 90% of cases [3,4]. EOC develops a thin lining in the epithelial tissue that covers the outside of an ovary. Among EOC, high-grade ovarian carcinoma (HGSOC) is known as the most frequent and aggressive form and is recognized as the primary cause of cancer-related deaths among gynecological cancer worldwide [4–7]. In contrast, low-grade serous ovarian carcinoma (LGSOC) is an infrequent form of EOC, accounting for less than 5% [8,9].

The standard treatment consists of optimal cytoreductive surgery with subsequent treatment with a combination of platinum and taxane-based chemotherapy [10,11]. In general, patients who positively respond to initial chemotherapy, classified as responders, show a favorable prognosis with a median survival rate exceeding four years. However, it has been known that one-third of HGSOC patients face disease progression or recurrence after initial treatment. These non-responders receive a second-line treatment not involving platinum agents [12–17]. Platinum-based chemotherapeutics, such as carboplatin and cisplatin, are agents that bind to DNA to form crosslinks with cancer cells, thereby inducing damage to the DNA that disrupts genome replication, transcription, and triggers cell apoptosis [18,19]. Paclitaxel, on the other hand, is a taxane that binds to microtubules, inhibiting its depolymerization [20,21]. The interference in the normal microtubule dynamics caused by paclitaxel during anaphase activates the spindle assembly checkpoint and thus induces mitotic arrest and apoptosis [21,22].

Prior studies have addressed drug-response prediction in OC. Lu et al. (2019) [23] focused on patients with EOC who experienced relapse after first-line chemotherapy. A drug response model was developed using the support vector machine algorithm. The training data were retrieved from the Cancer Cell Line Encyclopedia (CCLE) and were validated using the Cancer Genome Atlas (TCGA) and the GSE9891 datasets. The 10-gene predictive model demonstrated that the patients with high response have longer recurrence-free survival, suggesting potential benefits for other drugs for patients with partial or low response [23]. The study by Yu et al. (2016) [24] focused on analyzing tumor proteomic profiles and clinical characteristics of patients with SOC derived from the Clinical Proteomic Tumor Analysis Consortium. The drug response model was developed using the support vector machine algorithm and was evaluated using the leave-one-out cross-validation method. In addition, data-driven feature selection was performed to identify the predictive value of tumor proteomics profiles for platinum response. LASSO-Cox proportional hazards model selected features associated with ATP synthesis pathways and Ran GTPase binding which are indicative of platinum sensitivity. Overall, the study showed that proteomic profiles can predict drug response and provide information about biological processes affecting drug efficacy [24].

Our fundamental research questions were as follows: (1) what approaches can be implemented to increase the transferability of models across diverse datasets, (2) how do different feature selection methods influence the efficiency of machine learning models when applied to gene expression data, and (3) what biological pathways are linked to features discovered by machine learning models in predicting drug response to SOC patients? Herein, we applied machine learning models and gene expression profiles to identify precise multi-gene panels that can predict platinum-based chemotherapy response, with or without the addition of paclitaxel, in SOC patients. Our research study expands upon existing work in the realm of OC, incorporating progress in gene expression data analysis and drug response prediction. Our investigation involved rigorous pre-processing analyses and constructed a comprehensive approach via amalgamation of diverse feature selection methods instead of hinging on a singular method. This multi-method strategy yielded better machine learning outcomes and demonstrated biological relevance concerning SOC and the prediction of drug response.

## 2. Materials and Methods

**Figure 1.**
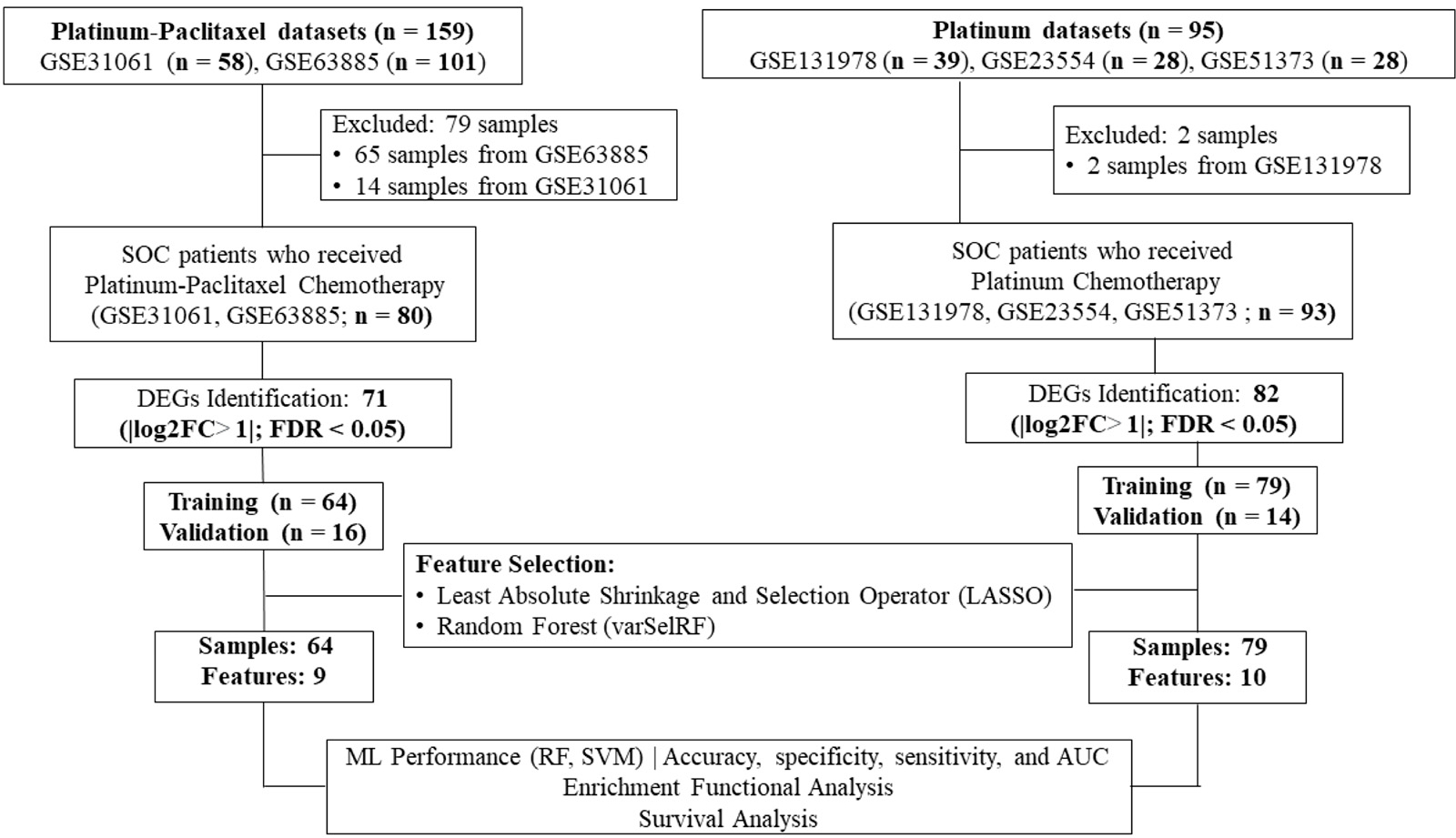
Workflow of the current study. Gene expression profiling datasets of human serous ovarian cancer tissues from the NCBI-GEO database were analyzed to identify differentially expressed genes (DEGs) using the robust multi-array average method in R. The LASSO and varSelRF feature selection methods were used to identify gene signatures related to each chemotherapy drug (i.e., platinum–paclitaxel or platinum-only). The performance of random forest and support vector algorithms as the machine learning model was evaluated. Functional enrichment analysis used the IPA online tool. Progression-free survival and overall survival analysis utilized the Kaplan–Meier plotter online tool.

The proposed machine learning framework consists of five main steps: data cleaning and pre-processing, feature extraction, feature selection, classification using machine learning classifiers, and biological significance analysis as shown in Figure 1.

### 2.1. Datasets

The set of binary files in a CEL format containing the ovarian cancer raw gene expression data were retrieved from the Gene Expression Omnibus (GEO) database using “GEOquery” R/Bioconductor library (http://www.ncbi.nlm.nih.gov/geo/, accessed on 6 June 2023). The terms “Serous-Ovarian-Cancer”, “Chemotherapy”, “Expression profiling by array”, and “Homo-sapiens” were used to find relevant experimental studies that examined the gene expression profiles of ovarian tumors in patients who either responded or did not respond to the drug. The chemotherapy regimens of interest are platinum-based chemotherapy. This methodology led to the identification of five datasets, including GSE131978, GSE23554, GSE51373, GSE63885, and GSE30161.

Clinicopathological information from the original studies was used for the analysis. The GSE131978 dataset contained samples from two different platforms, Affymetrix GeneChip Human Genome U133 Array (HG-U133A) and Affymetrix GeneChip Human Genome U133 Plus 2.0 Array (HG-U133Plus2). The samples were grouped based on the platform and were used as two separate datasets. The GSE131978—HG-U133A dataset contains 25 samples including 11 responders, 12 non-responders, and 2 samples with missing information. The two samples were removed from the dataset. The GSE131978—HG-U133Plus2 dataset, on the other hand, contains 14 samples including 7 responders and 7 non-responders. The GSE23554 dataset contains a total of 28 samples including 18 responders and 10 non-responders. The GSE51373 dataset contains a total of 28 samples including 16 responders and 12 non responders. The GSE63885 dataset contains a total of 101 samples including 65 responders and 10 non-responders. However, only 36 samples (24 responders and 12 non-responders) were used for this analysis. The remaining 65 samples were excluded from the study because these samples were not extracted from a serous ovarian tissue and/or received a different chemotherapy regimen. The GSE30161 dataset contains a total of 58 samples including 54 responders, 1 non-responder, and 3 samples with missing information. Only 44 samples (25 responders and 19 non-responders) were included in this study. The remaining samples were excluded because they were not extracted from serous ovarian cancer tissues. The samples were divided and organized into two groups based on the type of chemotherapy administered. The ratio of responders to non-responders was biased in some of the deposited GEO datasets including GSE63885 and GSE30161. Thus, the datasets for each chemotherapy regimen were combined for better outcomes.

Table 1 displays the GEO accession numbers of the expression datasets, along with the corresponding platform used for each dataset. In addition, the table includes the number of samples categorized as responders, non-responders, and the total number of samples. The reference manuscripts for each dataset utilized in this study are specified as well.

### 2.2. Inclusion and Exclusion Criteria

The following criteria determine the patients’ samples included in the study: (1) patients with serous ovarian cancer; (2) patients who underwent platinum-based chemotherapy; (3) a sample size of at least 10 for each dataset; (4) gene expression profiling datasets; and (5) available information about the drug response and/or recurrence and/or survival status. The exclusion criteria comprise: (1) datasets containing cell-line or xenograft samples; (2) samples with missing information about the drug type; and (3) samples with missing information about the drug response.

### 2.3. Machine Learning Framework

The machine learning framework followed methods to determine chemotherapy responders and non-responders from our previously published work [25].

#### 2.3.1. Data Pre-Processing, Quality Control, and Feature Extraction

The raw microarray expression data were retrieved from each GEO database. Certain samples were excluded from the raw non-normalized data because they contain missing information needed for the analysis. The Affymetrix data were analyzed using Guanine Cytosine Robust Multi-Array Analysis (GCRMA) from the Bioconductor package gcrma (version 2.44.0) [26] for the HG-U133A and HG-U133 Plus 2 platform types. The GCRMA algorithm conducts several data processing steps such as background correction, log2 transformation, quantile normalization, and summarization of probe sets into gene-level expression values [27,28]. The “nsFilter” function from the Bioconductor package genefilter (version 1.60.0) removed probes with minor sample variance and low median expression levels in the normalized dataset [29].

Quality control (QC) for each normalized dataset utilized the outlier removal strategy. The ArrayQualityMetrics R package (version 4.1.0) [30] was used for assurance and quality control of the microarray experiments. This approach enhances the effectiveness of meta-analysis and increases the ability to detect differentially expressed genes [31]. In the quality control procedure, samples that were identified as outliers were excluded from the relevant datasets. Following this, the raw data, which were devoid of any outliers, underwent a new round of normalization using the method elucidated in the preceding section. The normalized datasets were then used in further analysis.

All probes were mapped to their corresponding gene symbols, which serve as a universal identifier across platforms. The Official HUGO Gene Nomenclature Committee (HGNC) was employed as it is subject to rigorous curation and has been shown to improve the precision of scientific and public communication [32,33]. When there are multiple probes for a given gene symbol, the average expression value of all those probes was used to determine the expression level of that gene. Unannotated probes were disregarded from the analysis. The conversion process from probes to gene symbols was carried out using the R/Bioconductor package “org.Hs.eg.db” (version 3.14) [34]. Depending on the platform, the datasets were annotated with the R/Bioconductor packages hgu133a.db or hgu133plus2.db.

#### 2.3.2. Z-Score Transformation

Application of the Z-score transformation normalized the gene expression data using the “scale” function in R/Bioconductor package *stats.* This approach of normalization allows for consistent data across studies, allowing for direct comparison of microarray data regardless of differences in the initial hybridization intensities [35]. This approach has been used extensively in previous studies and has consistently shown effective performance in many applications [36].

#### 2.3.3. Batch Effect Correction

The issue of batch effects, which are systematic non-biological differences that can occur in multi-batch datasets due to variations in experimental conditions, was addressed in this study. To correct for batch effects and ensure the consistency of our different batches, we applied the ComBat algorithm from the ‘sva’ package in R [37]. This method used an empirical Bayes framework to adjust for both known and unknown batch effects, thus normalizing the data. The application of ComBat enables us to mitigate potential confounders, thereby improving the reliability and comparability of our findings. This step was crucial for the following analysis, ensuring that the biological interpretations derived from our data were not obscured by technical variability.

#### 2.3.4. Train/Test Split Using K-Fold Cross-Validation

To assess the machine learning model performance, the model first undergoes initial training using the training dataset, followed by an evaluation process on a validation set. The utilization of a cross-validation procedure is commonly used in situations where the dataset is constrained [38]. This process involves the iterative dividing of the data into distinct training and validation sets, which are subsequently used to train and assess the model, respectively. In this study, the training set split into 10 folds of approximately same size. An independent test dataset, on the other hand, refers to a distinct and independent set that has not been used in any capacity during the training and validation sets phases of the model. The R/Bioconductor *caret* package [39] was used to randomly divide the samples into training and test sets using the function “create folds”.

### 2.4. Differential Expressed Genes (DEGs)

Differential gene expression analysis used the ‘limma’ package in R, which was specifically designed for the analysis of gene expression data derived from microarray [40]. The ‘limma’ approach uses a linear model framework coupled with empirical Bayes methods to provide robust statistical inference even when dealing with complex experiments and relatively small sample sizes. After pre-processing and normalization of the data, application of ‘limma’ allows the identify genes that demonstrated statistically significant changes in expression between responders and non-responders. Visualization of differentially expressed genes with the Enhanced Volcano plot allows the effective display of the statistical significance against the fold change on a log scale, highlighting genes that are biologically interesting and potentially warrant further study. This visual representation was important in facilitating a clearer understanding of the key results from our differential expression analysis.

### 2.5. Feature Selection

In this study, the Variable Selection Random Forest (varSelRF) and Least Absolute Shrinkage and Selection Operator (LASSO) methods were combined to extract genes with the best predictive power. The capacity of these methods to concentrate on a small set of genes with strong prediction power led to their selection. Furthermore, they necessitate minimal parameter tuning, as the default settings frequently result in optimal performance.

#### 2.5.1. Least Absolute Shrinkage and Selection Operator (LASSO)

The LASSO technique is a form of regularization regression that is commonly used for the purpose of fitting a generalized linear model. The regression model is subjected to a penalty, specifically the L1 norm, which leads to the decrease in regression coefficients for variables that make minimal contributions towards zero. LASSO regression analysis was applied using the R/Bioconductor package glmnet (version 4.1) [41]. The LASSO method exhibits strong performance in situations where the dataset demonstrates a high number of dimensions and a low sample size. Numerous studies have consistently shown that this method exhibits significant potential as a promising model for feature selection [42,43].

The acquired results and the regression coefficients were used to establish a scoring system that attributes weights to the chosen signatures. The formula employed is as follows:

(1) $$Prediction\;Score=\sum_{i=1}^{n}\left({\text{β}}_{i}\times x_{i}\right)$$

The provided formula uses the variables “n” to represent the number of genes in the gene signature and “β” is utilized to denote the regression coefficient associated with the chosen gene signatures. The regression coefficient is obtained through LASSO logistic regression. Furthermore, the symbol “x” represents the expression value that corresponds to the selected signature.

#### 2.5.2. Variable Selection Random Forest (varSelRF)

The varSelRF method employs regression trees within the framework of random forest for the purpose of classification. The construction of the classification tree entails employing bootstrap samples, wherein each branch of the tree comprises a unique selection of candidate variables that are chosen randomly. The trees in varSelRF are generated using a method that integrates bootstrap aggregation (bagging) with feature selection within the random forest framework. Independent tree construction precedes the use of bagging and random variable selection methods to minimize inter-tree correlation, guaranteeing low-bias trees. The ntree parameter, representing the number of trees, was set to its default value of 2000, as was the mtry parameter, which determines the number of variables considered at each split [44].

### 2.6. Machine Learning Algorithms Performance

Two machine learning algorithms were used in this study: random forest and support vector machine. Random forest was applied via the R/Bioconductor package Random-Forest [45], while support vector machine was applied using the R/Bioconductor package e1071 [46]. Accuracy, sensitivity, and specificity were used as metrics to compare the efficacy of the models.

To mitigate the risk of overfitting and enhance the robustness of our model evaluation, we implemented model tuning using the ‘caret’ package in R. This involved a systematic approach known as grid tuning in R, which allows for an extensive search over a predefined space of hyperparameter values to identify the most robust model sittings. For random forest, we focused on optimizing the ‘mtry’, which dictates the number of variables randomly sampled as candidates at each split, and the ‘ntree’, which represents the number of trees grown. Specifically, a tuning grid with varying levels of ‘mtry’ chosen based on the number of predictors, and a fixed ‘ntree’ value to evaluate the impact of these parameters on model complexity and accuracy. For SVM, our approach was to refine the cost of constraints violation ‘C’ and kernel width parameters ‘sigma’, using a preset range of values to determine the optimal balance between model simplicity and error minimization. The ‘trainControl’ function facilitated 10-fold-cross-validation, ensuring that the chosen hyperparameters provided robust predictions across different subsets of data. This systematic tuning via ‘caret’ package not only helped in identifying the most effective model settings, but also significantly contributed to the reliability and validity of our predictive models, thus achieving a balance between complexity and generalization.

All computational methods and figure generation were implemented using R language programming version 4.0.1. on an Intel Core-i9 CPU with 16 GB of RAM, and 64-bit Windows 10 configuration. The computations for machine learning could be run in approximately 1 h.

### 2.7. Biological Pathway Analysis

The canonical pathway enriched by differential genes was performed using Ingenuity Pathway Analysis (IPA), a web-based software application (Ingenuity Systems http://www.ingenuity.com accessed on 6 June 2023) that identifies biological pathways and functions relevant to biomolecules of interest [47]. A core analysis was first constructed, and then a list of differential genes with their probe identification, FDR value and logarithmic fold change were uploaded to IPA [47]. Enrichment pathways of differential genes were generated based on the Ingenuity Pathway Knowledge Data Base.

### 2.8. Validation of the ExpressionAnalysis

In this study, the Gene Expression Profiling Interactive Analysis (GEPIA2) online tool, accessible at http://gepia2.cancer-pku.cn/ (accessed on 6 June 2023) was used to conduct an analysis on the relevance of gene signatures in association with overall survival (OS) of patients diagnosed with ovarian cancer. The survival curve in the survival analysis was derived using the Kaplan–Meier method using the online tool accessible at https://kmplot.com/analysis/ (accessed on 6 June 2023). The log-rank test was used to assess the statistical significance of the observed difference. Statistical significance was determined by assessing the *p*-value, which was deemed significant if it was less than or equal to 0.05. Additionally, the median was employed as a cut-off criterion. Survival curves were generated incorporating hazard ratios (HR) and log-rank *p*-values for analysis.

## 3. Results

### 3.1. Data Extraction and Bactch Effect Analysis

Responder (49) and non-responder (31) samples meeting the inclusion criteria from the Affymetrix GeneChip human Genome U133 Plus 2.0 arrays platform (HG-U133_Plus_2) with a 54, 676 probes from the two GEO datasets, GSE301061 and GSE15888 were used for the platinum–paclitaxel analysis. The 54,676 hybridized probes utilized in these datasets targeted a total of 20,864 unique gene symbols, out of which the two datasets shared 15,167 gene symbols.

The same type of analyses was performed on datasets that contain gene expression profiles of SOC patients who received platinum-only. Responders (52) and non-responders (41) samples from the Affymetrix GeneChip human Genome U133 Plus 2.0 arrays platform (HG-U133_Plus_2) with a total of 54,676 probes as well as samples from the Affymetrix GeneChip human Genome U133a with 22,284 probes, were selected from the GEO datasets GSE31978 and GSE23554. Among these datasets, there were 6261 shared gene symbols.

**Figure 2.**
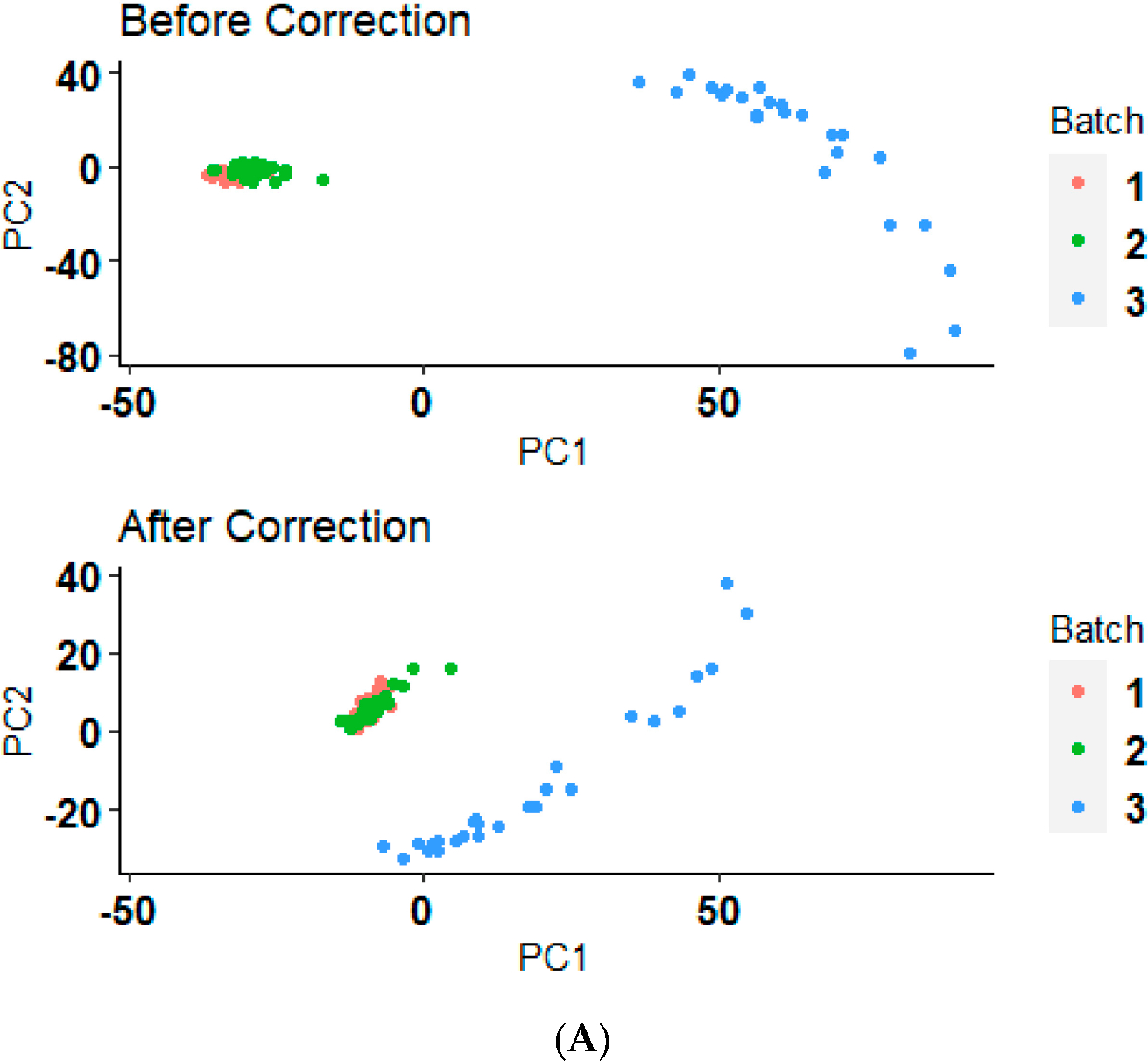

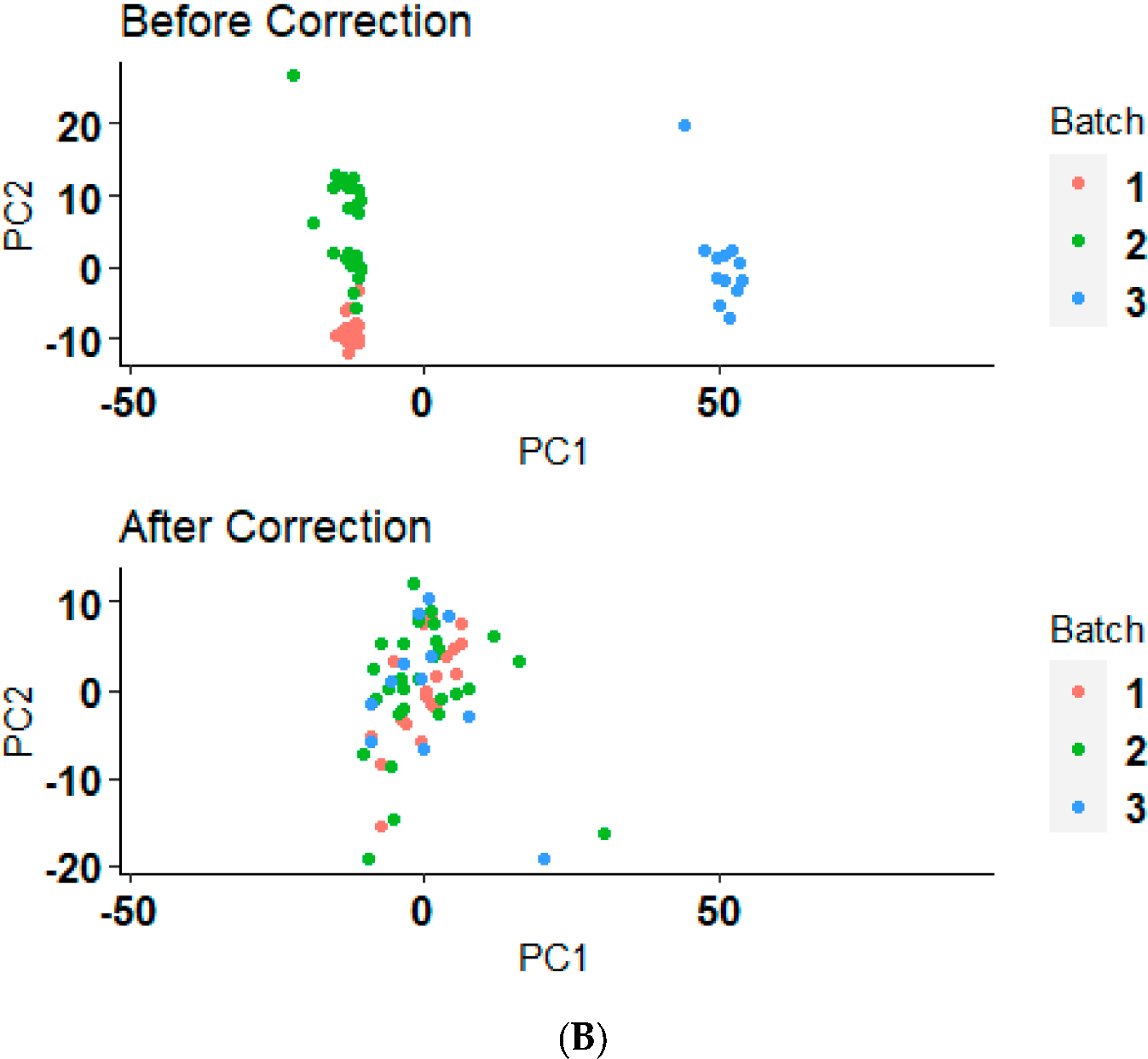
Before and after batch correction PCA clustering plot. (**A**) The PCA results before and after applying a batch correction method on serous ovarian cancer samples who received the platinum–paclitaxel drug. (**B**) The PCA results before and after applying a batch correction method on serous ovarian cancer samples who received the platinum-only drug.

Interpretation of the batch effects and their correction is crucial for assessing the reliability of biological conclusions drawn from multi-batch datasets. The principal component analysis (PCA) illustrated in Figure 2A,B demonstrates the distribution of data samples before and after a batch correction method application. Initially, the samples, as represented by the “Before” group, show a clear clustering pattern that likely reflects batch effects rather than underlying biological or experimental conditions (Figure 2A,B). After the application of the batch correction technique, as seen in the “After” group, these clusters appear to have converged, indicating a reduction in batch effects (Figure 2A,B). This reorganization not only highlights the effectiveness of the ComBat correction from the ‘sva’ package, but also reinstates confidence in the biological insights derived from the data. The reduction in batch-associated variance and the enhanced alignment of clusters along biological variables highlights the robustness of our analytical approach, confirming that the observed differences in gene expression are due to underlying biological effects rather than technical artifacts. This correction essentially allows us to proceed with downstream analyses and interpretations with a higher degree of reliability, focusing on biological variations that are truly pertinent to our study.

Following the pre-processing steps, the merged dataset was split into training and validation sets using a ten-fold cross-validation approach. The training set of platinum–paclitaxel consisted of 64 samples, comprising 39 responders and 25 non-responders. The validation set, on the other hand, included 16 samples, with 10 responders and 6 non-responders. On the other hand, the training dataset for platinum-only comprised 74 samples, with 42 samples classified as responders and 32 samples classified as non-responders. The validation set comprised a total of 18 samples, consisting of 9 responders and 9 non-responders (Figure 1).

### 3.2. Differential Expressed Genes Identified from the Platinum–Paclitaxel and Platinum-Only Data

Differentially expressed genes (DEGs) between the samples of responders and non-responders of the training set were determined using the “limma” package in R. The *p*-values were adjusted using the Benjamini–Hochberg (BH) method to control the false discovery rate (FDR), and a cut-off threshold of an adjusted *p*-value < 0.05 was applied.

**Figure 3.**
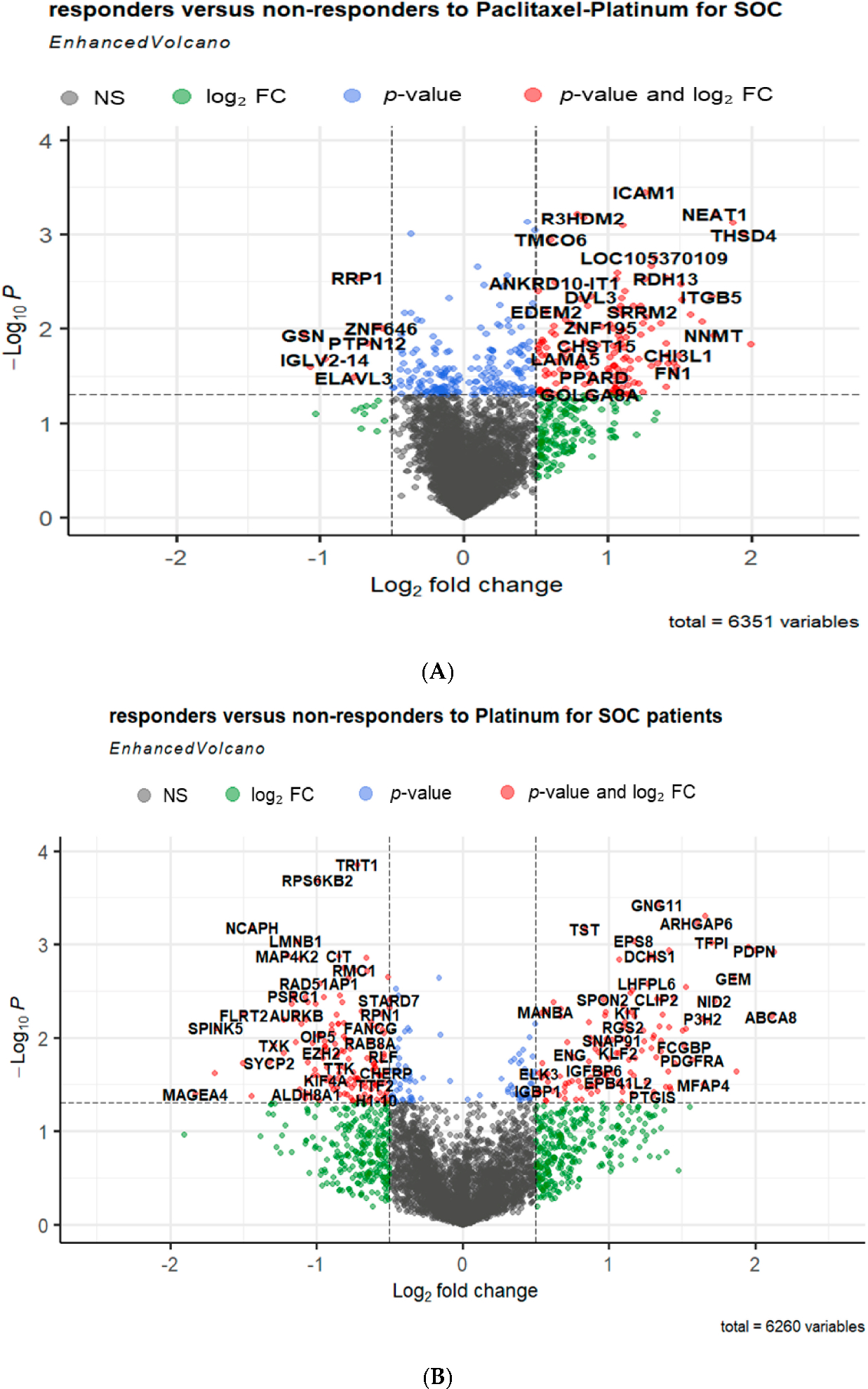
Volcano plots showing the distribution of the gene expression fold changes in serous ovarian cancer patients who received either (**A**) platinum–paclitaxel or (**B**) platinum-only treatment. The *x*-axis of the plot represents the log_2_ fold change in gene expression [log_2_ fold change = log_2_ $\left(\left(X_{i}^{D}/X_{i}^{C}\right)\right)$ where $X_{i}^{D}$ and $X_{i}^{C}$ are the average intensities of the gene of responders and non-responders, respectively], indicating the direction and magnitude of change. The *y*-axis displays the negative logarithm of the adjusted *p*-value, emphasizing the statistical significance of each gene’s expression difference. Red dots represent genes with a statistically significant increase or decrease in expression, indicated by a log2 fold change (log2FC) greater than 1 or less than −1 and adjusted *p*-value less than 0.05. Blue dots indicate genes with statistically significant adjusted *p*-value less than 0.5, but with a log2 FC that do not reach the set cut-offs for up-or downregulation. Green dots show genes that, while not meeting the stringent criteria for up-or downregulation, display a noteworthy fold change or *p*-value, suggesting potential biological significance. Grey dots correspond to genes that do not meet the significance threshold for differential expression, with fold changes and *p*-values that do not reach the set cut-offs for up-or downregulations.

In total, 71 DEGs were identified between the tissue samples of non-responders and responders in patients with SOC who received platinum–paclitaxel treatment (Supplement Table S1). Among these DEGs, 69 genes were found to be upregulated, while only 2 genes were downregulated (Figure 3A). However, 82 DEGs were identified when comparing the tissue samples of non-responders and responders in patients diagnosed with SOC who underwent platinum treatment. Within the set of DEGs, it was observed that 58 genes were found to be upregulated, whereas 25 genes were downregulated (Figure 3B).

### 3.3. Gene Signatures Identified from LASSO and varSelRF Feature Selection Methods

To filter out the feature genes based on OC-related DEGs, two feature selection methods, LASSO regression and varSelRF, were used.

**Figure 4.**
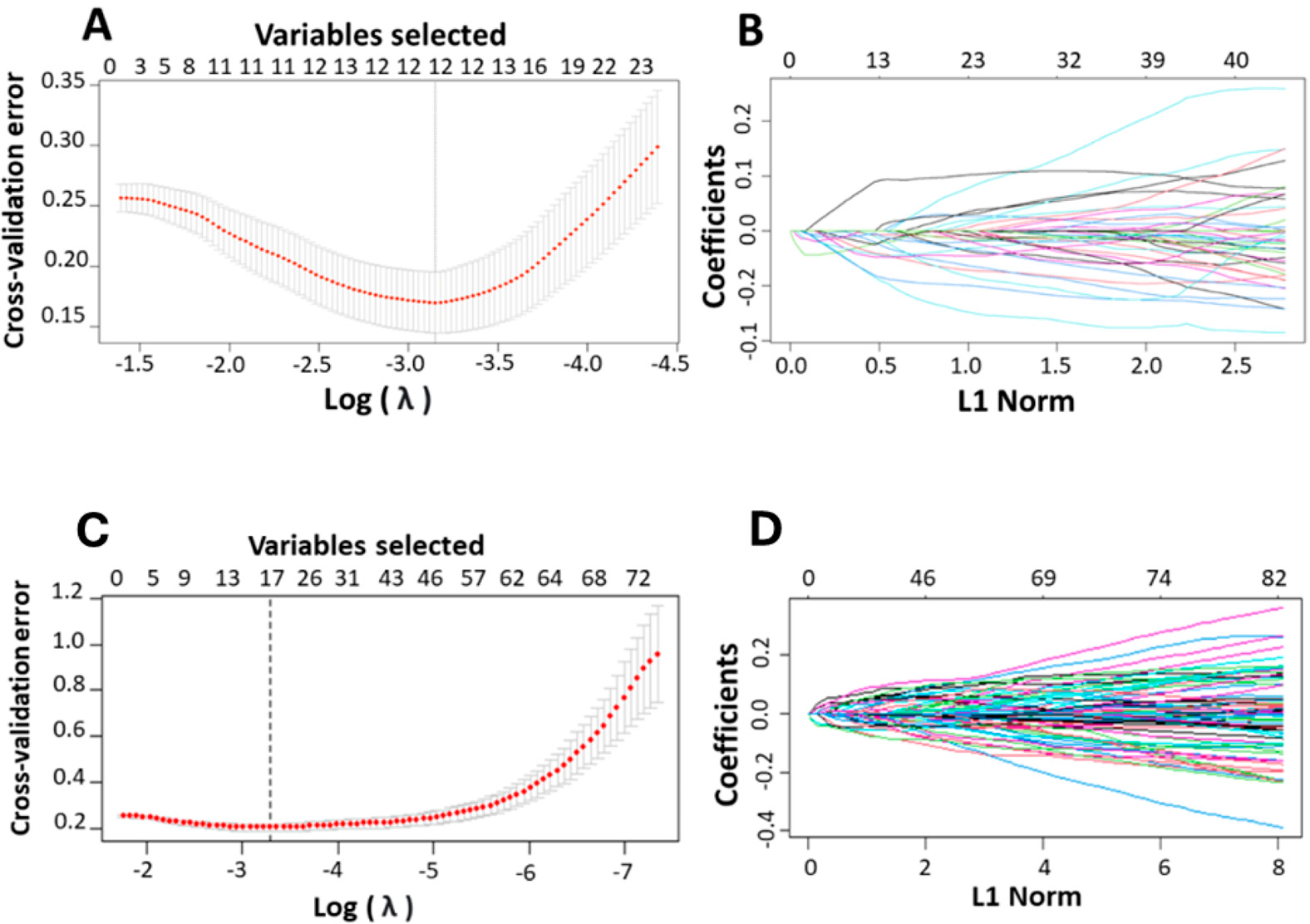
Identification of the relevant genes associated with ovarian cancer and platinum-based drug using LASSO. (**A**,**C**) The cross-validation error plots in a LASSO model. The plots provide insights into the model’s performance across different levels of complexity represented by varying values of the regularization parameter, lambda. The *x*-axis represents the lambda values on a logarithmic scale helping to visualize the wide range of lambda values explored during the model fitting process. The error bars on the mean cross-validation error curve show the standard error for different lambda values, indicating the variability in model performance across complexities. Smaller error bars suggest greater confidence in the error estimates at those lambda values. A vertical line drawn at the lambda value corresponding to the minimum average cross-validation error. This line identifies the optimal level of model complexity, balancing bias, and variance to achieve the best predictive performance. (**B**,**D**) The partial likelihood deviation plotted against lambda using the LASSO model. These plots illustrate the trajectory of each predictor’s coefficient as the regularization parameter (L1 Norm or lambda) changes, helping to identify which predictors are most influential in the model. Each line in the plot represents the coefficient of a predictor variable in the model, plotted against varying values of lambda. As lambda increases, the plot shows how each coefficient is shrunk towards zero. The entry or exit of lines across the zero line indicates when predictors are being added to or removed from the model highlighting their relative importance.

For LASSO analysis, ten-fold cross-validations was performed to calculate the cross-validation error and to determine the optimal lambda (λ) value. The λ value corresponding to the minimum cross-validation error, denoted as λmin, was selected as the optimal λ. A dotted vertical line was plotted at the λ value chosen through 10-fold cross-validation. Out of 71 DEGs identified from the training set of platinum–paclitaxel, 12 non-zero coefficients (genes) were associated with minimum cross-validation error of 0.038 (Figure 4A,B). These 12 genes are: ICAM1, TUBB2A, GLDC, PLAU, AURKA, SRRM2, DCHS1, NEAT1, MXRA5, NRBP2, GSN, and MUC16. The prediction score is computed using Equation (1).

Following same concept on the platinum-only training data, out of 82-identified DEGs, 17 non-zero coefficients (genes) were associated with minimum cross-validation error of 0.0371 (Figure 4C,D). The 17 gene signatures include NBL1, FCGBP, LMNB1, FLRT2, NUAK1, MAP4K2, SPINK5, LRRC17, SYCBP, TXK, IL12A, CCN2, CORO2B, CLIP2, HSPA2, PAQR4, and TFPI. The prediction score is computed using Equation (1). As mentioned in the methodology section, the varSelRF method was used to confirm the results achieved by LASSO methods.

For the platinum–paclitaxel data, eleven feature genes were selected with the varSelRF method including, AEBP1, GSN, ICAM1, NEAT1, TUBB2A, PLAU, MXRA5, MUC16, GLDC, AURKA, and CD81. Nine feature genes were defined by overlapping the genes derived from these two feature selection methods, including Intercellular Adhesion Molecule 1 (ICAM1), Tubulin Beta 2A Class (TUBB2A), Glycine Decarboxylase (GLDC), Plasminogen Activator, Urokinase (PLAU), Aurora Kinase (AURKA), Nuclear-Enriched Abundant Transcript 1 (NEAT1), Matrix Remodeling Associated 5 (MXRA5), Gelsolin (GSN), and Mucin-16 (MUC16).

For the platinum-only training data, 13 feature genes were selected with the varSelRF method including LMOD1, FCGBP, TFPI, NUAK1, SPINK5, LRRC17, FLRT2, CCN2, IL12A, HSPA2, CDC20, MAP4K2, and FOXM1. A total of ten feature genes were defined by overlapping the genes derived from these two feature selection methods, including Fc Gamma Binding Protein (FCGBP), Tissue factor pathway inhibitor (TFPI), NUAK Family Kinase 1 (NUAK1), Cell Division Cycle 20 (CDC20), Leucine-Rich Repeat Containing 17 (LRRC17), Fibronectin Leucine-Rich Transmembrane Protein 2 (FLRT2), CCN2, Interleukin 12A (IL12A), Heat Shock Protein Family A (Hsp70) Member 2 (HSPA2), Forkhead Box M1 (FOXM1), and mitogen-activated protein kinase kinase kinase kinase 2 (MAP4K2).

### 3.4. Validation of the Gene Signatures Using the GEPIA Database

**Figure 5.**
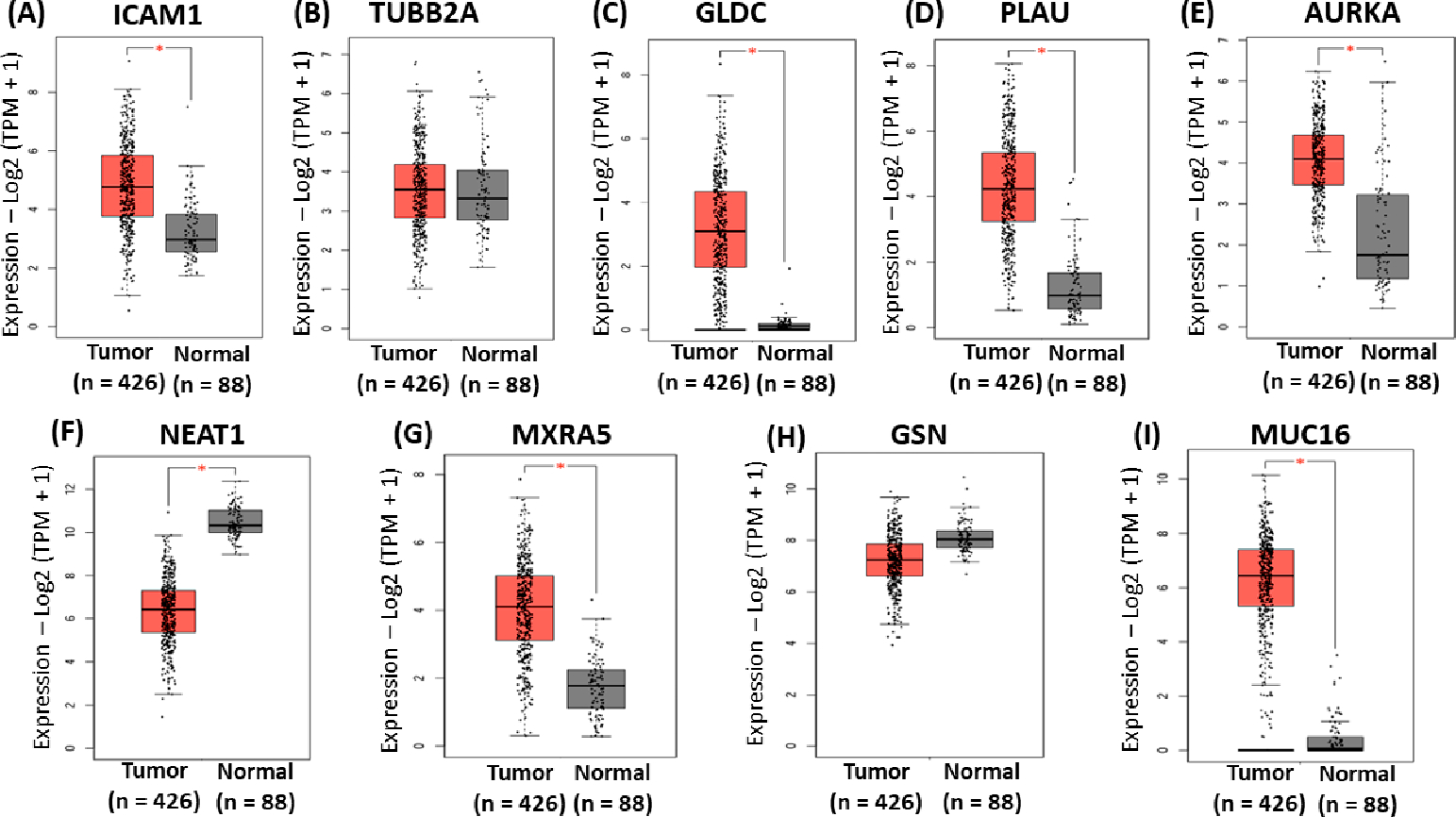
Validation of the identified gene signatures associated with platinum–paclitaxel using GEPIA2. Comparison of expression of (**A**) ICAM1 (**B**), TUBB2A (**C**), GLDC (**D**), PLAU, (**E**) AURKA, (**F**) NEAT1, (**G**) MXRA5, (**H**) GSN, and (**I**) MUC16 between ovarian cancer tissues and normal tissues. The red asterisk symbol above the boxplots indicates statistical significance between tumor and normal tissues. A single asterisk represents a *p*-value less than 0.05.

The mRNA expression of the gene signatures in normal and OC tissues was analyzed using GEPIA2 software accessible at http://gepia2.cancer-pku.cn/ to validate the expression of the identified gene signatures. Based on datasets from databases such as TCGA and GTEx, the results reveal that the mRNA levels of ICAM1, GLDC, PLAU, AURKA, MXRA5, and MUC16 were significantly higher in OVCA than in normal tissues (Figure 5A,B–E,G,I). In contrast, the mRNA level of NEAT1 was significantly lower in OVCA than in normal tissues (Figure 5F). There was no statistically significant difference in the expression levels of TUBB2A and GSN between tumor and normal tissues (Figure 5B,H).

**Figure 6.**
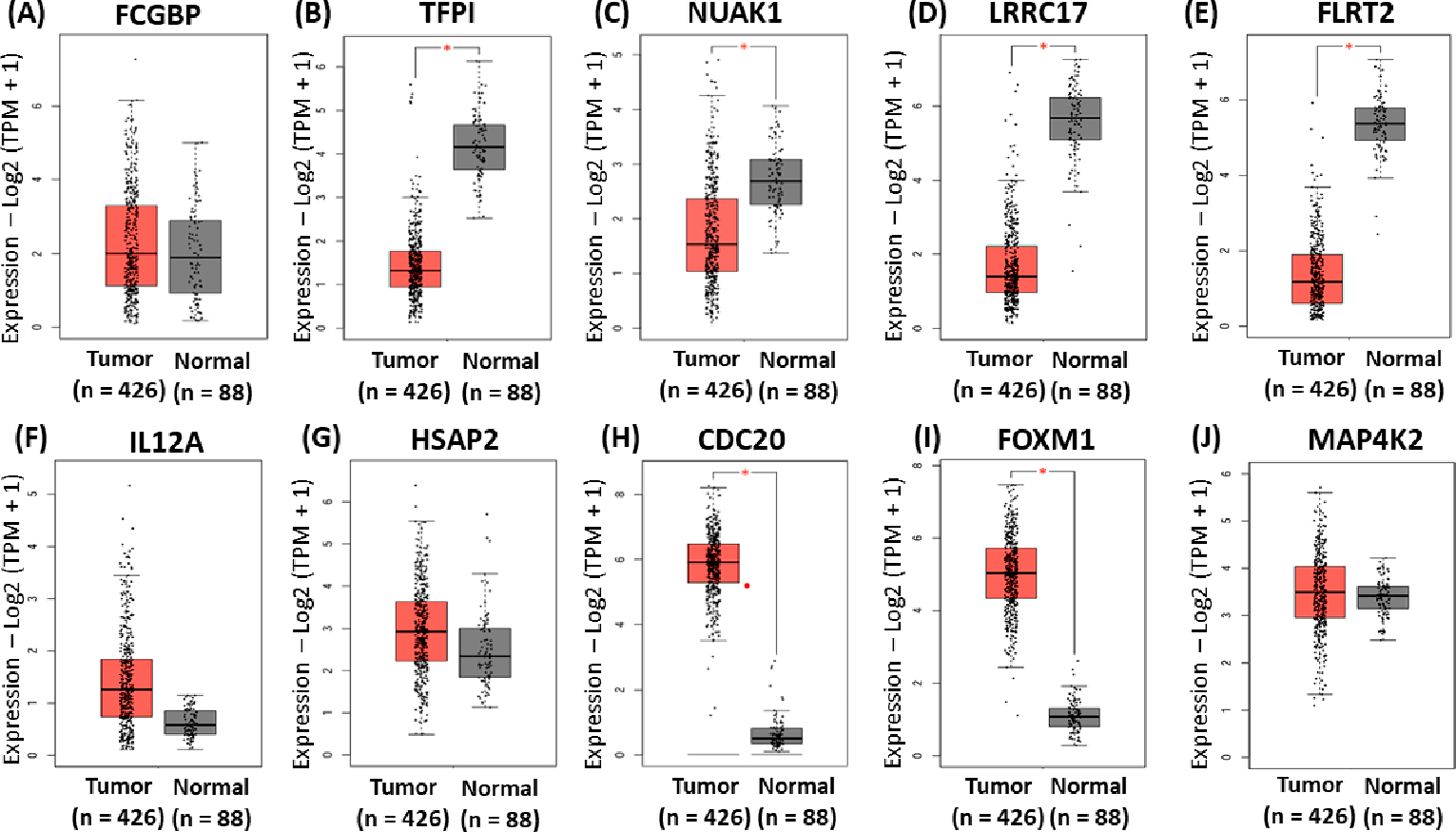
Validation of the identified gene signatures associated with platinum-only using GEPIA2. Comparison of expressions of (**A**) FCGBP (**B**), TFPI (**C**), NUAK1 (**D**), LRRC17, (**E**) FLRT2, (**F**) IL12A, (**G**) HSPA2, (**H**) CDC20, (**I**) FOXM1, and (**J**) MAP4K2 between ovarian cancer tissues and normal tissues. The red asterisk symbol above the boxplots indicates statistical significance between tumor and normal tissues. A single asterisk represents a *p*-value less than 0.05. The red dot represents an outlier, indicating that the expression level of a particular sample is much higher or lower than the rest of the data in the tumor group.

The same analysis was performed to validate the gene signatures identified for the platinum-only data. The mRNA expression of the gene signatures in normal and OC tissues was analyzed using GEPIA2 software to validate the expression of the identified gene signatures. These results indicated significantly higher mRNA levels of CDC20, and FOXM1 in OVCA compared to normal tissues (Figure 6C–E,G,I). In contrast, there were significantly lower mRNA levels of TFPI, NUAK1, LRRC17, and FLRT2 in OVCA compared to normal tissues (Figure 6F,H). Furthermore, FCGBP, MAP4K2, IL12A, and HSPA2 showed similar expression levels in ovarian cancer compared to normal tissues (Figure 6A,F,J).

### 3.5. Machine Learning Classification Performance

The performance evaluation of the models was conducted on both the training and validation sets using metrics such as accuracy, sensitivity, specificity, and AUC. Random forest was the best-performing machine learning algorithm, with the SVM algorithm following closely behind (Table 2).

For the platinum–paclitaxel training set, the random forest algorithm achieved an accuracy of 1 with a 95% confidence interval (CI) ranging between 0.92 and 1. The sensitivity and specificity were equal to 0.98 and 0.95, respectively. Support vector machine, on the other hand, achieved an accuracy of 0.94 with a 95% CI ranging between 0.82 and 0.98. The specificity and sensitivity were equal to 0.91 and 0.96, respectively. About the platinum-only training set, random forest algorithm achieved an accuracy of 0.99 with a 95% CI ranging between 0.95 and 1. The sensitivity and specificity were equal to 1 and 0.99, respectively. Support vector machine, on the other hand, achieved an accuracy of 0.97, with a 95% CI ranging between 0.90 and 0.96. The specificity and sensitivity were equal to 0.97 and 0.96, respectively (Table 2).

For the platinum–paclitaxel validation set, random forest algorithm had an accuracy of 0.91 with a 95% CI ranging between 0.77 and 0.96. In addition, sensitivity, specificity, and area under curve (AUC) were equal to 0.86, 0.92, and 0.91, respectively. The support vector machine algorithm obtained an accuracy of 0.91 which was equal to the value obtained from the random forest algorithm. In terms of sensitivity, specificity, and AUC, they were equal to 0.82, 0.92, and 0.90, respectively. On the other hand, for the platinum-only validation set, the random forest algorithm had an accuracy of 0.95 with a 95% CI ranging between 0.89 and 0.97. In addition, sensitivity, specificity, and area under curve (AUC) were equal to 0.95, 0.94, and 0.94, respectively. The support vector machine algorithm obtained an accuracy of 0.93 which was equal to the value obtained from the random forest algorithm. In terms of sensitivity, specificity, and AUC, they were equal to 0.95, 0.90, and 0.93, respectively (Table 2).

### 3.6. Biological Significance of the Identified Gene Signatures

To acquire an enhanced comprehension of the biological relevance of the gene signatures that were differentially expressed in SOC, the IPA software version (24.0) was used to subject the genes to analysis identifying the molecular processes that were significantly implicated. The ingenuity pathways knowledge base has provided pertinent insights on canonical pathways, diseases, and disorders, as well as molecular and cellular functions.

In terms of the canonical pathways, the nine genes are involved in glycine cleavage complex (*p*-value = 3.49 × 10^−3^), germ cell–Sertoli cell junction signaling (*p*-value = 4.28 × 10^−3^), tumor microenvironment pathway (*p*-value = 4.73 × 10^−3^), and tumoricidal function of hepatic natural killer cells (*p*-value = 1.39 × 10^−2^). In regard to the 10 genes associated with platinum–paclitaxel, they are involved in the following canonical pathways: the NOD1/2 signaling pathway (*p*-value = 2.65 × 10^−3^), hepatic cholestasis (*p*-value = 2.71 × 10^−3^), natural killer cell signaling (*p*-value = 2.91 × 10^−3^), protein ubiquitination pathway (*p*-value = 5.45 × 10^−3^), and extrinsic prothrombin activation pathway (*p*-value = 6.64 × 10^−3^).

In terms of molecular and cellular functions, IPA demonstrated that the nine aforementioned genes are involved in cell–to–cell signaling and interaction (*p*-value range 1.09 × 10^−2^–1.63 × 10^−7^), cellular assembly and organization (*p*-value range 1.10 × 10^−2^–3.13 × 10^−5^), cellular function and maintenance (*p*-value range 8.13 × 10^−3^–3.13 × 10^−5^), cellular development (*p*-value range 9.8 × 10^−2^–4.22 × 10^−5^), and cellular growth and proliferation (*p*-value range 9.8 × 10^−3^–4.22 × 10^−5^). In regard to the 10 genes associated with platinum–paclitaxel, they are involved in the cell cycle (*p*-value = 4.2 × 10^−2^–1.51 × 10^−7^), cellular development (*p*-value = 4.40 × 10^−2^–1.51 × 10^−7^), cell death and survival (*p*-value = 4.44 × 10^−2^–6.73 × 10^−5^), and cellular movement (*p*-value = 4.76 × 10^−2^–9.20 × 10^−5^).

**Figure 7.**
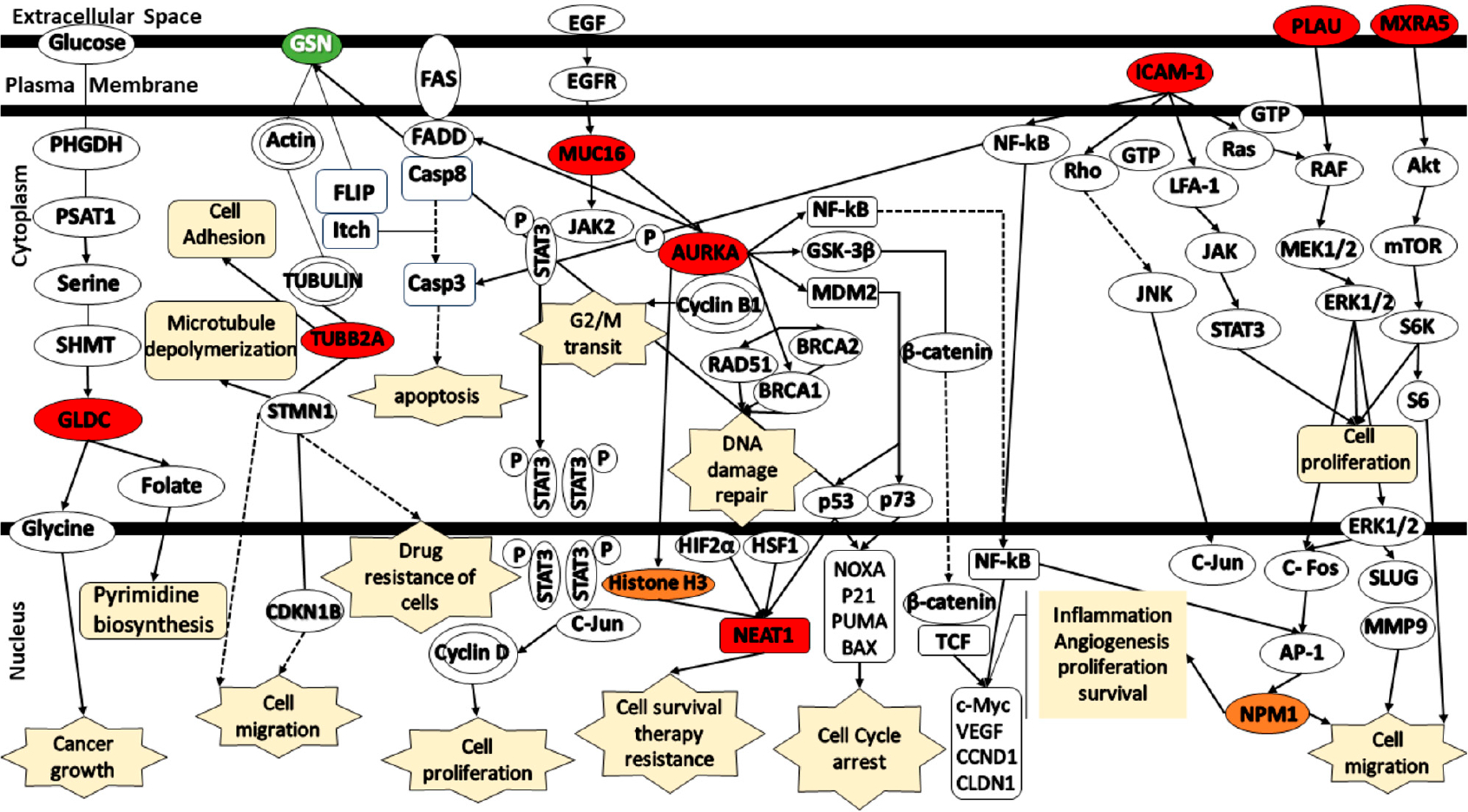
Schematic representation of the signaling pathways for the gene signatures predicting in the response of serous ovarian cancer patients to platinum–paclitaxel. (Green color—under expression; red color—over expression; orange color— activation; dashed lines—indirect relationship; solid lines—direct relationship). Abbreviations: AURKA, Aurora Kinase A; AP-1, Activator Protein 1; GSN, Gelsolin; GLDC, Glycine Decarboxylase; ICAM1, Intercellular Adhesion Molecule 1; MXRA5, Matrix Remodeling Associated 5; MUC16, Mucin-16; NEAT1, Nuclear-Enriched Abundant Transcript 1; NPM1, Nucleophosmin 1; PLAU, Urokinase-Plasminogen Activator; TUBB2A, Tubulin Beta 2A; TP53, Tumor Protein 53.

Furthermore, the generation of a schematic representation that illustrates the relationship between the gene signatures in different signaling pathways used the IPA library. Figure 7 illustrates the pathways associated with the identified platinum–paclitaxel genes. GLDC was found to be involved in cancer growth through the PSAT1/Serine/SHMT/GLDC/Glycine complex. TUBB2A is associated with cell migration through STMN1/CDKN1B or cell adhesion via action. GSN was indirectly involved in the induction of apoptosis through the FLIP/Itch/Casp8 complex. MUC16 facilitates cell proliferation through the JAK2/STAT3/c-Jun/Cyclin D complex. It was also associated with G2/M cell cycle transition through the AURKA/Cyclin B1 complex. AURKA participates in many different cascades including NFκB, GSK3β, MDM2/p53, BRCA1/2, and RAD51. It is involved in cell cycle, inflammation, angiogenesis, cell proliferation and survival. NEAT1 is involved in cell cycle, cell survival and therapy resistance through Histone H3, p53, HIF2α, and HSF1. ICAM-1 participates in cell migration and proliferation via NFκB, NPM1, Pho/JNK, GTP/LFA-1/JAK/STAT3, and Ras/RAF/MEK/ERK activates the Raf/MEK/ERK pathway which is responsible for cancer cell proliferation and migration. PLAU has been observed to be associated with the Akt/mTOR/S6k pathway, which also activates cancer cell proliferation and migration.

**Figure 8.**
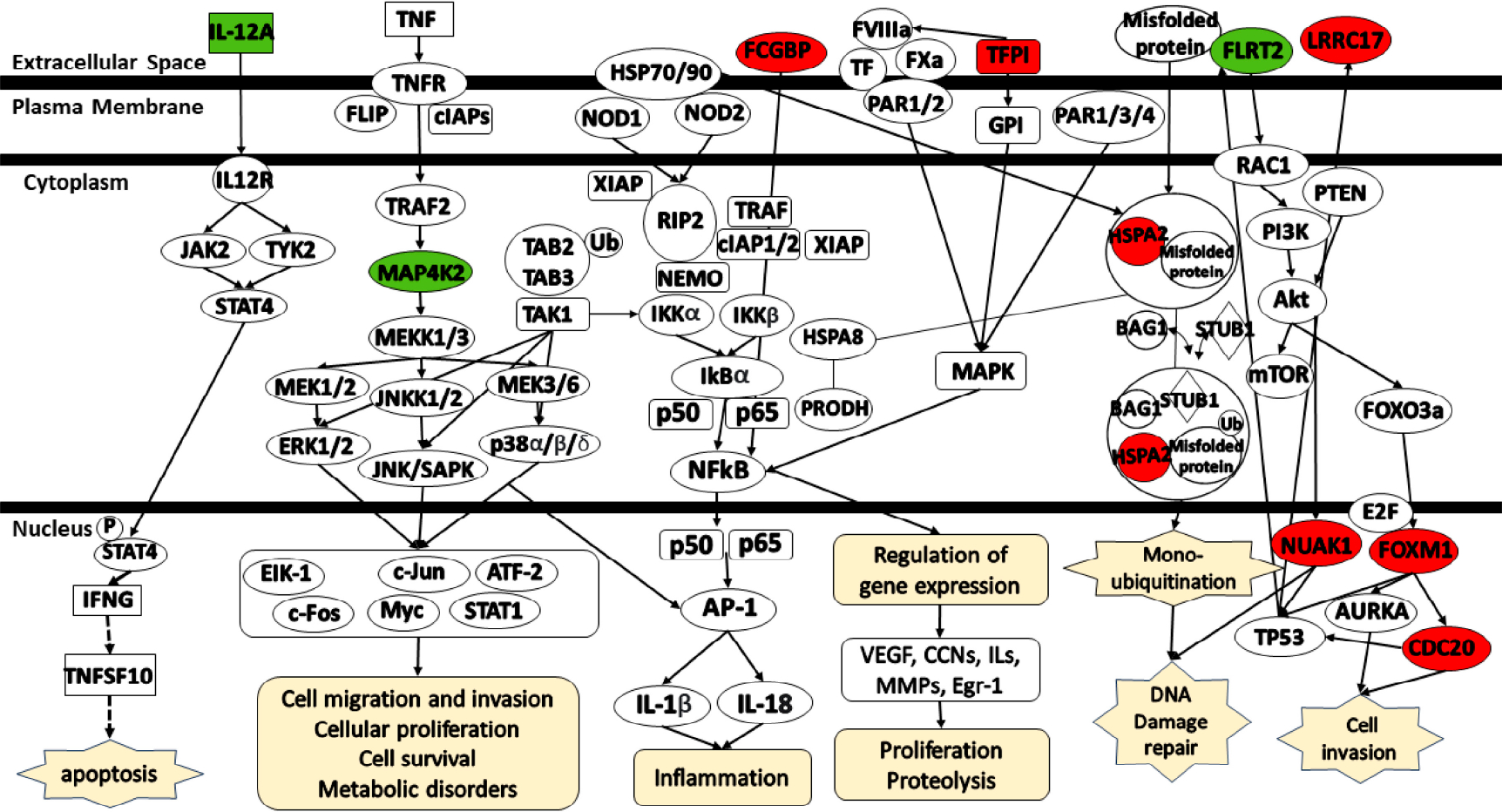
Schematic representation of the signaling pathways for the gene signatures predicting the response of serous ovarian cancer patients to platinum-only. (Green color—under expression; red color—over expression; dashed lines—indirect relationship; solid lines—direct relationship). Abbreviations: CDC20, Cell Division Cycle 20; HSPA2, Heat Shock Protein Family A (Hsp70) member 2; IL-12A, Interleukin 12 A; FCGBP, Fc Gamma Binding Protein; FOXM1, Forkhead box M1; FLRT2, Fibronectin leucine-rich transmembrane protein 2; LRRC17, Leucine-rich repeat containing 17; MAP4K2, MAPK Kinase Kinase Kinase 2; NUAK1, NUAK Family Kinase 1; TFPI, Tissue factor pathway inhibitor.

Figure 8 illustrates the pathways associated with the identified platinum–paclitaxel genes. IL12a mediates signaling via either Jak2 or Tyk2, along with p-STAT4, IFNG, and TNFSF10, thereby contributing to the induction of apoptosis. MAP4K2 is activated through various transduction signals, including those initiated by TNF and TRAF2. MAP4K2 mediates signaling via MEKK1/3 activating the MEK/ERK, JNK/SAPK, and p38 α/β/δ pathways. These pathways activate multiple signals including EIK-1, c-Jun, ATF-2, c-Fos, Myc, and STAT1 which involve in cell migration and invasion, cellular proliferation, cell survival, and metabolic disorders. TFPI was found to be associated with FVIIIa, FXa, TF, and PAR1/2. TFPI mediates signaling via GPI and MAPK which, in turn, mediates NFκB signaling. HSPA2 is directly connected to HSP70/90 which, in turn, have an association with NOD1/2. NOD1/2 serves as a mediator for various signaling cascades that promote NFκB signaling. MEK/ERK, JNK/SAPK, p38 α/β/δ, and NFκB pathways activate the AP-1 which subsequently promotes inflammation. NFκB pathway also stimulates proliferation and proteolysis by modulating the expression of key factors, including VEGF, CCNs, ILs, MMPs, and Egr-1. HSPA2 participates in DNA damage repair by modulating the expression of BAG1, STUB1, and the ubiquitination of misfolded protein. FLRT2 acts as a mediator by modulating the expression of RAC1/PTEN/PI3K/Akt/mTOR. Akt is involved in the activation of FOXO3a which, in turn, mediates the signaling of FOXM1 and CDC20. Both FOXM1 and CDC20 play a role in cell invasion. Akt also participates in the activation of NUAK1. Finally, both LRRC17 and NUAK1 affect TP53 expression.

### 3.7. Survival Analysis Using Kaplan–Meier

Survival analysis can assess the impact of gene signatures on the survival of SOC patients. Plotting the Kaplan–Meier survival curves for the gene signatures using the web-based curator considering lower and higher expression of genes and using the default parameters of a multiple hypothesis testing statistical method (*p* = 0.05). The analysis followed KM plotter guidelines and opted for a Bonferroni correction threshold under 10% FDR to calculate significant analysis. Application of the Affymetrix ID of each gene further explored the prognostic potential of the gene signatures by assessing their correlation with histology type, grade, and chemotherapy type. A total of 1657 OC samples collected from GEO and TCGA databases were found in the KM Plotter database. The correlation between gene signatures expression and the clinical parameters was determined using univariate and multivariable Cox regression. The analysis was restricted to SOC samples who underwent debulking surgery and received either platinum–taxol or platinum-only chemotherapy. Further filtering of the data separated LGSOC (grade 1 and 2) from HGSOC (grade 3).

In regard to platinum–paclitaxel-related genes, ICAM-1 overexpression was associated with better OS in LGSOC, but no significant difference in LGSOC. TUBB2A, GLDC, and AURKA were associated with worse OS in all grades of SOC. While overexpression of PLAU and MXRA5 was only associated with worse OS in LGSOC, there was no significant difference in expression in HGSOC. In addition, there was no significant association between the expressions of NEAT1, GSN, and MUC16 and OS in all grades of SOC (Table 3).

With genes related to platinum-only treatment, the overexpression of HSPA2, NUAK1, LRRC17, and FLRT2 was associated with worse OS in all grades of SOC. FCGBP was associated with worse OS in HGSOC, but no significant difference in expression in LGSOC. On the other hand, there was no significant association between the expressions of TFPI, FOXM1, MAP4K2, CDC20, and IL12A and OS in all grades of SOC (Table 4).

### 3.8. Machine Learning Model Application to Predict Effectiveness of Alternate Chemotherapy Regimen

The genes successfully classifying responders and non-responders for platinum–paclitaxel were different from the genes successfully classifying responders and non-responders for platinum-only suggesting different underlying mechanisms. It is possible that patients who did not respond to the combination of platinum–paclitaxel might respond to platinum-only treatment, and vice versa. Interestingly, the application of the random forest model for the platinum–paclitaxel dataset to cases of SOC treated with the platinum regimen, suggest that 34 of 93 (36.55%) SOC patients who did not respond to platinum–paclitaxel would respond to platinum-only treatment (Table 5). While this seems counter intuitive, there is evidence in previous studies, described in the Discussion section, that suggests that resistance can arise with the combination therapy that does not occur with the monotherapy case.

## 4. Discussion

This study applied machine learning models and gene expression profiles to identify precise multi-gene panels that can predict the response to platinum-based chemotherapy, with or without the addition of paclitaxel, in SOC patients. Indeed, this study demonstrated promising outcomes as a clinical indicator, showing a high level of accuracy. The random forest and support vector machine classifiers accurately classified responders’ and non-responders’ tumor samples in the GEO SOC validation sets with 91% accuracy for platinum–paclitaxel and 95% and 93% accuracy for platinum-only, respectively. The findings demonstrated that features identified by machine learning can distinguish resistant from sensitive tumors to the chemotherapy regimen. Our results were similar to the results of two previous studies [16,48]. Their models also achieved accuracy above 90% in both the training and validation sets.

### 4.1. Mechanisms od Platinum Agents

The mechanism of action of the platinum agents (i.e., cisplatin or carboplatin) is conditioned by the covalent binding of these molecules to DNA making DNA crosslinks and, eventually, inhibition of cell cycle and cell proliferation [49,50]. Some factors contributing to platinum resistance include overexpression of multidrug resistance proteins, advancement of DNA repair mechanisms, degradation, and deactivation of intracellular thiols [51]. The activation of cellular protective responses inhibits cell cycle progression to facilitate the repair of cisplatin-induced DNA damage [52]. Recognition of platinum-induced DNA damage occurs by diverse cellular mechanisms, including the MRE11-RAD50-NBS1 complex, hMSH2 of the mismatch repair (MMR) complex, the nonhistone chromosomal high-mobility groups 1 and 2 proteins (HMG 1/2), and the transcription factor “TATA-binding protein” (TBP) [53]. These specific proteins recognize the damage and transmit signals to proteins such as p53, p73, and MAPK, leading to apoptosis and cell death [53]. MAPK signals (i.e., extracellular signal-related kinases (ERKs), c-Jun N-terminal kinases (JNKs), p38 kinases) play an important role in platinum-induced effects, with controversial data regarding their involvement in apoptosis. ERK activation induces p53 phosphorylation, resulting in cell cycle arrest, DNA damage repair, and activation of pro-apoptotic genes, ultimately leading to apoptosis. In addition, it has been shown that cisplatin induces p18 stabilization, which is a substrate of p38 kinases, increasing p53’s ability to activate the transcription of proapoptotic genes such as PUMA and NOXA [53].

### 4.2. Mechanisms of Taxane Agents

The mechanism of action of the taxane agents is the induction of cellular death by binding to tubulin and inhibiting the disassembly microtubules required for chromosome segregation and cell division. In addition, taxane treatment inhibits cell proliferation, induces apoptosis, and triggers diverse stress responses such as autophagy, senescence, and inflammation through complex mechanisms [53]. Similar to platinum-based chemotherapies, paclitaxel resistance develops in cancer cells via the efflux of paclitaxel out of the cells. Resistance to taxane agents can be attributed to PI3K/AKT hyperactivation including loss of function in PTEN and increases in anti-apoptotic Bcl-2 family members. The activation of these molecules can overpower the anti-proliferative signals leading to the upregulation of factors involved in cell proliferation and migration [54].

### 4.3. Pathogenic Role of Genes Identified in Platinum–Paclitaxel Study

The genes identified in this study play crucial roles in the pathogenesis of serous-ovarian cancer and chemoresistance, offering significant biological insights and potential clinical applications. For instance, for platinum–paclitaxel, our study identified nine gene signatures associated with platinum–paclitaxel resistance, including ICAM1, TUBB2A, GLDC, PLAU, AURKA, NEAT1, MXRA5, MUC16, and GSN. These genes are involved in pathways that have been previously reported to be associated with chemoresistance in different cancers, including epithelial ovarian cancer. Particularly, we found out in our study that overexpression of ICAM-1, MXRA5, AURKA, and NEAT1 are associated with the activation of NPM1, Histone H3, and TP53 in patients with serous ovarian cancer who received the platinum–paclitaxel chemotherapy regimen. A previous study showed that there is a link between an overexpression of nuclear NPM1 protein, chemoresistance, and poor outcomes for women diagnosed with HGSOC through the DNA repair function of APE1 and Ref-1 proteins [55]. APE1/Ref-1-NPM1 proteins are linked to cancer aggressiveness, which supports the idea that interfering with the APE1/Ref-1-NPM1 interaction might enable improved sensitization of cancer cells to chemotherapy [55]. Studies also showed that overexpression of ICAM-1 and AURKA increases the level of histone H3 [56,57]. The enhancer of zest homolog 2 (EZH2), a family member of the histone methyltransferases (HMT), can promote the cancer development through the catalyzation of the trimethylation of lysine at position 27 of histone H3, resulting in the suppression of downstream tumor suppressor genes [58]. Yang et al. (2020) reported that EZH2 was overexpressed in cisplatin-resistant OC cells compared to sensitive OC cells leading to blockage of cell death and proliferation of OC cells. ICAM-1 upregulation by the activation of multiple pathways including PKCα-p38-SP-1, JAK, PI3K, AKT, and NFκB has been observed previously [59]. The association of ICAM-1 overexpression was reported to be associated with reduced progression-free survival in SOC patients treated with platinum–paclitaxel compared to those treated with platinum-only, suggesting that patients with high ICAM1 expression might be resistant to Paclitaxel [59]. AURKA was found to be amplified in more than 15–25% of OC cell lines and primary tumors and to cause resistance to cisplatin by activating proteins such as p-eIF4E, c-MYC, HDM2, BRCA1/2 [60,61]. In addition, clinical data showed that patients with BRCA1/2 mutations respond better to cisplatin, leading to the hypothesis that AURKA has a synergistic effect with BRCA1/2 in platinum resistance [60]. AURKA has been identified to regulate many signaling pathways, such as the PI3K/Akt, mTOR, β-catenin/Wnt, and NFκB pathways, and tumorigenesis requires interactions among multiple signaling pathways [62]. The elevated MXRA5 was reported to be associated with tumor angiogenesis [63,64]. Bioinformatics studies and protein chip analyses identified an association between overexpressed MXRA5 and PI3K-Akt-mTOR cascade in pancreatic cancer cells [64]. MXRA5 is also upregulated in breast cancer and was found to be important for the EMT progression and matrix remodeling [64,65]. Previous studies showed that silencing NEAT1 inhibits the invasion of OC cells in vitro and attenuates tumor growth in vivo [66–68]. The knockdown of NEAT1 was associated with the increase in cisplatin–taxol sensitivity in MDA-MB-231 OC cells [69]. This study also reported that elevated NEAT1 expression and paraspeckle formation form part of such malignancy-associated stress response pathways such as p53 [69].

Furthermore, other previous studies showed a linkage between TUBB2A, PLAU, GLDC, and MUC16 and chemoresistance. TUBB2A, essential component of microtubules, related to growth, infiltration, and drug resistance in several different malignancies [70]. GLDC was found to enhance glycolysis and is highly expressed in tumor-initiating-cells in non-small cell lung carcinoma [71]. Interestingly however, Shin et al. (2018) reported that GLDC is downregulated in paclitaxel-resistant OC cells and was suggested to be associated with OC chemoresistance [72]. Kwon et al. (2015) reported that mitochondrial glycine synthesis, closely coupled to serine via a single reversible step catalyzed by serine hydroxy methyltransferase (SHMT), was associated with rapid cancer cell growth [71]. Further research on GLDC and drug resistance in OC is required to validate the results. The upregulated PLAU was associated with platinum–paclitaxel drug resistance and worse OS in LGSOC, but no significant difference in expression was observed in OS for HGSOC in our analysis. A recent study reported that PLAU overexpression promotes progression in ESCC and tumors including breast, bladder, and lung cancer [73]. Another study reported that the downregulation of PLAU reduces the EMT-related genes expressed in the oral squamous cell carcinoma (OSCC) cell line leading to cessation of cell migration and invasion [74]. PLAU promotes ESCC proliferation and tumor growth by activating the MAPK pathway [73]. MUC16 stimulates cell adhesion, growth, and metastasis, and evading attacks from natural killer cells aiding cancer cell progression [75]. A previous study reported that silencing MUC16 increased the sensitivity of OVCAR-3 cells to cisplatin and doxorubicin but not to paclitaxel [76]. Another study found that the overexpression of MUC16 induces breast cancer cell proliferation via its interaction with the non-receptor tyrosine kinase JAK2, and this interaction mediates phosphorylation of transcription factor STAT3, which may transactivate c-Jun for Cyclin D1 expression [77]. Furthermore, decreased MUC16 expression results in an accumulation of breast cancer cells at the G2/M phase of the cell cycle via Cyclin B1 and phosphorylation of AURKA, which in turn leads to apoptosis of breast cancer cells through JNK signaling [77]. GSN participates in multiple important cellular signaling for motility, apoptosis, proliferation, differentiation, epithelial mesenchymal transition, and carcinogenesis phenotypes [78]. GSN plays roles as both the effecter and inhibitor of apoptosis, which underlines its association in a wide variety of cancer types [78]. A recent study by Arentz et al. (2023) [79] found that overexpression of GSN was significantly associated with HGSOC patients treated with chemotherapy. Interestingly, another recent study contradicts our findings and the findings of Arentz et al. (2023), demonstrating that the expression and secretion of GSN were higher in chemo-resistant OC cells than in chemo-sensitive OC cells [80]. The supporting study by Onuma et al. (2022) [81] suggested that higher levels of GSN prevent cisplatin from dissociating GSN from the FLIP-ITCH complex, thus preventing caspase-3 and -8 activation and caspase-mediated GSN cleavage and thereby inhibiting apoptosis in chemo-resistant OC cells. However, further analysis is needed to understand the role of GSN in OC.

### 4.4. Pathogenic Role of Genes Identified in Platinum-Only Study

For the platinum-only drug, our study identified ten gene signatures associated with platinum–paclitaxel resistance including FCGBP, HSPA2, TFPI, NUAK1, LRRC17, FOXM1, CDC20, FLRT2, MAP4K2, and IL12A. Our analysis showed that these genes are associated with NOD1/2, natural killer, ubiquitin–proteosome, and tissue-factor-activated complex pathways. NOD1/2 act as an oncogene in ovarian cancer by upregulating immune-related pathways such as the RIPK2/NFκB signaling pathway [82]. Upregulation of these immune-related pathways seems to modulate several stress response systems eventually disrupting both proliferation and cellular migration via PI3K/Akt/mTOR, MAPK, TNF, and p53 signaling pathways [83]. For instance, inhibition of Akt confers resistance to cisplatin through p53– (FLICE)-like inhibitory protein (FLIP) interaction and FLIP ubiquitination, which was attenuated by p53 silencing [84,85]. For instance, our study showed that the overexpression of NUAK1, LRRC17, FOXM1, and CDC20 as well as the downregulation of FLRT2 are associated with DNA repair pathway as well as tumor invasion pathways. Two previous studies have shown that NUAK1 overexpression was associated with platinum and taxane resistance in SOC patients. In addition, these studies reported a direct interaction between NUAK1/LKB1 and p53 pathway, as well as the NFκB pathway, particularly in HGSOC cells [86,87]. A recent study found that LRRC17, an inhibitor of the receptor activator of the NFκB ligand (RANKL), is a potent prognostic factor in SOC, demonstrating a significant correlation between the overexpression of LRRC17 and poor OS in SOC patients. In addition, the study suggested that overexpressed LRRC17 can inhibit chemotherapy-induced apoptosis in SOC [88]. The upregulated CDC20 was linked to platinum-only drug resistance in our analysis. CDC20 is one of the regulators of spindle checkpoint [89]. A previous study reported that CDC20 was remarkably upregulated by the knockdown of p53 [89]. The overexpression of CDC20 was significantly associated with SOC compared to the other types of OC. After silencing CDC20, EOC cell proliferation and migration decreased, and apoptosis increased [90]. FOXM1, on the other hand, has emerged as a multifunctional oncoprotein and a robust biomarker of poor prognosis in many human malignancies [91]. The FOXM1 transcriptional pathway was aberrantly activated in over 85% of cases and was rendered the second most frequent molecular alteration in HGSOC, second only to TP53 mutations [91]. Downregulation or inactivation of the p53 and Rb pathways results in the activation of the E2F1 transcription factor, which directly upregulates FOXM1 gene expression by binding to its promoter. These findings establish that p53 and Rb pathway dysregulation is a key contributor to FOXM1 overexpression in OC [91]. Finally, possible association of FLRT2 down-regulation with the process of ovarian and uterine cancers due to downregulated expression has been suggested [92]. Its biological function was verified only in prostate cancer and breast cancer [92], but its role in the tumorigenesis of OC remained unclear. Further research is needed to determine the role of FLRT2 in SOC.

Natural killer (NK) cells, which are lymphocytes of the innate immune system involved in the early defenses against foreign cells, express an array of activating cell surface receptors that can trigger cytolytic programs, as well as cytokine or chemokine secretion [93]. A recent study showed that carboplatin, one of the platinum agents, increased HLA-E, nectin-4, HLA-ABC, and CD111 expression in HGSOC cell lines, which was associated with an inhibitory NK receptor ligand phenotype [94]. Of these, nectin-4 was reported to have a role in HGSOC metastasis and chemotherapeutic resistance [94]. For instance, IL12A, which was identified in our study, was reported to play a critical role in the regulation of early inflammatory responses and promotion of the Thy1-type repertoire [95]. IL-12A stimulates T-cells and NK cells to secrete IFN-γ and increases the proliferation and cytolytic activity of these cells. IL-12A was reported to be an effective anti-cancer agent against various experimental malignancies [95].

The ubiquitin–proteasome pathway plays an important role in the regulation of cellular proteins involving cell cycle control, transcription, apoptosis, cell adhesion, angiogenesis, and tumor growth [96]. Various ways that the ubiquitin pathway is involved in OC, such as modulating the ovarian-cancer-related gene BRCA1 and tumor suppressor p53, and interfering with the ERK pathway, the cyclin-dependent cell cycle regulation process, and ERBB2 gene expression [96]. HSPA2, one of stress-non-inducible and least characterized members of the HSPA family (HSP70), is ubiquitous in various types of cancer cells [97]. HSP70 overexpression has been linked to ovarian cancer aggressiveness [98]. HSP70 has been shown to support tumor growth and invasion in EOC via modulating several cellular events including cell cycle, apoptosis, and epithelial mesenchymal transition pathways [98]. FCGBP has been found to be downregulated in many cancers including ovarian cancer [99]. It plays an important role in anti-inflammation and cell protection in epithelium cells as well as cell adhesion [99]. Cell adhesion occurring in the vasculature of specific organs is an essential step in cancer metastasis [99].

The tissue-factor-activated fVII (fVIIa) complex is an essential initiator of the extrinsic blood coagulation process [100]. Interactions between cancer cells and immune cells via coagulation factors and adhesion molecules can promote progression of cancer, including EOC [100]. TF, fVII, intercellular adhesion molecule-1 (ICAM-1), and multiple pro-inflammatory cytokines can be induced in response to hypoxia in EOC cancer cells at the gene expression level, leading to the autonomous production of the TF–fVII complex [100,101]. TFPI, a novel serodiagnostic marker for EOC, inhibits blood coagulation induced by tissue factor [102]. The diminished expression of TFPI could result in activated factor Xa and increase factor Xa-PAR2 signaling [103]. Various studies have suggested that therapeutic strategies that target an increase in the expression of TFPI could inhibit tumor angiogenesis, growth, and metastasis [103].

### 4.5. Translations Potential

Finally, previous studies showed that 20 to 30% of OC patients fail to respond to the platinum–paclitaxel combination [104]. When patients progress on platinum–paclitaxel chemotherapy, it remains uncertain whether resistance has developed to one or both of the drugs, despite being labeled as platinum–paclitaxel-resistant [104]. Our findings were partially supported by previous studies [104,105]. Judson et al. evaluated the efficacy of combination drug therapy on cisplatin-resistant OC cells and found that cisplatin exerts mechanistic dominance over paclitaxel when human OC cells are simultaneously exposed to combination of cisplatin and paclitaxel [104]. This dominance adversely affects cisplatin-resistant cells by inhibiting paclitaxel-induced apoptosis [104]. Thus, suggesting that patients might derive significant benefits from a trial of paclitaxel alone as a second-line regimen in cases where initial treatment with cisplatin/paclitaxel has proven ineffective [104]. A recent study from Choi et al. supported the findings of Judson et al. [105]. They tested the effects of combined cisplatin and paclitaxel on cisplatin-resistant oral squamous cell carcinoma cells and found that cell growth was more inhibited by paclitaxel alone than combination therapy [105]. In addition, their study further suggests that the overexpression of FOXM1 protein by cisplatin makes it difficult to overcome drug resistance to cisplatin and causes resistance to paclitaxel, which can impact the effectiveness of combination therapy [105]. Unfortunately, our data did not contain patients who received paclitaxel-only treatment to validate the results of these studies. Nonetheless, our findings concord with the results of Choi et al. As shown in Figure 6, our data demonstrate that overexpression FOXM1 was associated with resistance to platinum in patients with SOC. Thus, we speculate that this might be the underlying reason for the non-response observed in patients subjected to the platinum–paclitaxel model. Nevertheless, further clinical validation would be needed before this could influence clinical care.

From a clinical perspective, the identified gene signatures hold significant promise for improving serous ovarian cancer diagnosis and treatment. They can offer profound insights for enhancing diagnostic accuracy and tailoring personalized treatment strategies. These gene signatures could serve as biomarkers for the early detection of serous ovarian cancer, particularly in conditions where early intervention can significantly alter clinical outcomes. In addition, they have substantial potential in prognostic evaluations, providing clinicians with the ability to predict the progression of serous ovarian cancer and patient responses to platinum-based chemotherapy more precisely. Our findings indicate that certain genes within these signatures are associated with resistance to platinum-based chemotherapy, a common treatment regimen for ovarian cancer. This resistance often leads to treatment failure and poor prognosis. By identifying patients who are genetically predisposed to this resistance, clinicians can avoid ineffective platinum-based therapies and instead opt for alternative treatment protocols that might be more effective. This preemptive approach not only spares patients from the side effects of ineffective treatment but also significantly reduces treatment costs and duration. Furthermore, understanding the mechanisms behind this resistance opens up ways for the development of new drugs aimed at modifying the expression or function of these resistant genes. For instance, novel inhibitors could be designed to target specific proteins encoded by the genes within the resistant signature, potentially restoring sensitivity to platinum-based treatments. This could revolutionize treatment protocols and improve survival rates for patients who would otherwise have limited options.

### 4.6. Algorithm Selection Choices

We chose the LASSO and varSelR feature selection methods because of their complementary strengths in handling high-dimensional data, which is a common characteristic of gene expression datasets. LASSO is particularly effective due to its ability to perform both variable selection and regularization simultaneously. This method helps in enhancing the prediction accuracy while reducing the complexity of the model by shrinking coefficients of less important variables to zero, thus effectively selecting a smaller subset of more relevant features. The ability of LASSO to impose a constraint on the model parameters makes it particularly suitable for models that suffer from multicollinearity which is a frequent issue in genomic data [106]. On the other hand, varSelRF is a non-linear approach using the random forest algorithm. Unlike LASSO, which is based on linear model, varSelRF is capable of capturing complex interactions between features, which is often required in understanding biological systems. The random forest algorithm provides an intrinsic ranking of feature importance based on how much feature decreases the purity of the node, allowing for effective identification of relevant biomarkers that might be missed by linear methods [107]. We aimed to use the linear and non-linear strengths of these methods by employing both LASSO and varSelRF, respectively. This approach allowed us to capture a broad spectrum of informative features in our analysis, thereby enhancing the biological relevance and robustness of the identified gene signatures. The combination of these methods ensures a more comprehensive analysis that could be achieved through a single method, especially in datasets where the underlying biological relationship can be complex and non-linear. We strongly believe that this strategy has provided a balanced and rigorous approach to feature selection, offering a substantial justification for the selection and application of these specific methods in our study.

### 4.7. Data Analysis Challenges and Limitations

We encountered challenges due to imbalanced classes and missing data that had to be addressed. To ensure the rigor and transparency of our study, we adhered closely to the PRISMA diagram for systematic reviews and meta-analysis, applying strict inclusion and exclusion criteria throughout the data selection process. The inclusion criteria required that datasets exclusively contain information pertinent to the cancer type and histology, specifically serous ovarian cancer and must be tissue samples. In addition, it was crucial that the datasets included detailed information about the experimental platform used and the drug responses outcomes. Stages of cancer were also an integral factor in our analysis. Samples lacking any of this essential information were excluded from further analysis. Following this systematic approach, the study was structured into two subsections based on the treatment regimen: one focusing on datasets of patients who received a combination of platinum–paclitaxel drugs, and the other on those treated with platinum-only. From the beginning, these datasets demonstrated an inherent imbalance in class sizes between responders and non-responders within each treatment category. To address this imbalance, we employed several strategies to mitigate overfitting without resorting to data balancing methods such as oversampling, which we avoided due to the inherent risk of introducing artificial bias and overfitting. Oversampling the minority class can lead to models that perform well on repeated synthetic samples but fail to generalize to new, read-world data. Instead, we focused on alternative approaches. First, we used algorithms less sensitive to class imbalance, such as tree-based methods including random forest, which inherently manage class disparity by focusing on data structure rather than frequency. Secondly, we adopted robust evaluation metrics such as specificity, sensitivity, and area under curve that provide a clearer indication of model performance across unbalanced classes. We also implemented stratified cross-validation to ensure representative class distribution in each fold, enhancing model evaluation and stability. In addition, regularization techniques such as LASSO were applied to limit model complexity and prevent overlearning from the majority class. Finally, ensemble methods such as random forest were used to sequentially correct errors from previously built models, placing greater emphasis on previously misclassified instances, often from the minority class. These strategies collectively helped in reducing the risk of overfitting while improving model robustness and accuracy across our unbalanced datasets.

A strength of this study is that previous prediction studies included patients with varying clinical characteristics and histological types of OC, which made generalizability difficult. In this study, to ensure the validity of our research, only patients diagnosed with SOC were included. One limitation of the study is the sample size of the overall data. To mitigate the risk of overfitting that can be caused due to the small sample size, we have implemented several strategies. The K-fold cross-validation across the training and validation set was applied to ensure that the performance of the models is consistently evaluated against multiple data splits, enhancing the generalizability of our findings. Feature selection methods were also applied to ensure that only relevant predictors were included, minimizing the chance of the model capturing irrelevant variability. We plan to further validate our model using a larger dataset to provide a more robust test of its predictive power. This will help in refining our model and potentially reducing the observed differences in performance between training and validation sets.

Despite the potential constraints posed by the sample size, it is crucial to recognize the importance of accurately defining outcomes and ensuring homogeneity within the population when constructing prediction models. These factors must take precedence in order to produce reliable and valid results.

## 5. Conclusions

In conclusion, the current study found gene signatures capable of making high-accuracy prediction of the response to platinum-based chemotherapy in patients with serous ovarian cancer. This machine learning approach predicts a useful approach for improving drug treatment outcomes for cancer patients. This approach has significant potential for integration into clinical practice after additional clinical validation.

Analysis of the gene signatures gives the following insights into the important mechanisms for platinum–paclitaxel resistance in both low- and high-grade serous ovarian cancer. The non-responders to the drug seem to have genes including ICAM1, TUBB2A, GLDC, PLAU, AURKA, NEAT1, MXRA5, GSN, and MUC16 that promote cancer growth and cell proliferation through dysregulation of JAK2, STAT3, MAPK, AKT, and mTOR as well as DNA damage via BRCA1/2 and TP53. These genes are associated with pathways such as glycine cleavage complex and tumor microenvironment known to be linked to chemoresistance in many cancers including OC.

The analysis of gene signatures associated with platinum-only creates insights into the important mechanisms for platinum resistance in both low- and high-grade SOC. The change in expression in the following genes, FCGBP, TFPI, NUAK1, LRRC17, FLRT2, IL12A, HSPA2, CDC20, MAP4K2, and FOXM1, results in cell proliferation and invasion via aberration of JAK2, STAT4, and MAPK as well as apoptosis inhibition via TP53, AURKA, and NFκB. These genes have been previously reported to be associated with pathways involved in chemoresistance in OC including NOD1/2, natural killers (NK), ubiquitin–proteasome, and the tissue-factor-activated fVII complex.

Finally, our analysis as well as previous research demonstrated that overexpression of FOXM1 was associated with resistance to platinum in patients with SOC. Thus, we speculate that this might be the underlying reason for the non-response observed in patients subjected to the platinum–paclitaxel model. Nevertheless, further clinical validation would be needed before this could influence clinical care.

## Supplementary Material

Supplement

## Figures and Tables

**Table 1.** Description of each dataset for two different chemotherapy regimens.

GEO Accession	Platform	Number of Samples	Title/Description

GSE131978	GPL96 ^1^	25 (11 responders + 12 non-responders + 2 NA)	FXYD5 (dysadherin) upregulation predicts shorter survival and reveals platinum resistance in high-grade serous ovarian cancer
	GPL570 ^2^	14 (7 responders + 7 non-responders)	

GSE23554	GPL96 ^1^	28 (18 responders + 10 non-responders)	Ovarian Cancer Dataset

GSE51373	GPL570 ^2^	28 (16 responders + 12 non-responders)	Gene expression data from high grade serous ovarian cancer

GSE63885	GPL570 ^2^	101 (24 responders + 12 non-responders + 65 NA)	Gene expression profiling in ovarian cancer

GSE30161	GPL570 ^2^	58 (25 responders + 19 non-responders + 14 NAs)	Genomic Multivariate Predictors of Response to Adjuvant Chemotherapy in Ovarian Carcinoma: Predicting Platinum Resistance

1 GPL96 = Affymetrix GeneChip Human Genome U133 Array (HG-U133A),

2 GPL570 = Affymetrix GeneChip Human Genome U133 Plus 2.0 Array (HG-U133Plus2).

**Table 2.** Classification method performance on training and validation sets of SOC patients who were treated either with platinum-paclitaxel or platinum-only.

	Model		Accuracy (95% CI) ^1^	Sensitivity	Specificity	AUC ^2^

Platinum-Paclitaxel	Training (n = 64)	Random Forest	1 (0.92,1)	0.98	0.95	1
		Support Vector Machine	0.94 (0.82, 0.98)	0.91	0.96	0.94

	Validation (n = 16)	Random Forest	0.93 (0.85, 0.95)	0.90	0.94	0.93
		Support Vector Machine	0.93 (0.85, 0.95)	0.89	0.92	0.93

Platinum-Only	Training (n = 74)	Random Forest	0.99 (0.95, 1)	1	0.99	0.99
		Support Vector Machine	0.97 (0.90, 0.96)	0.97	0.96	0.97

	Validation (n = 19)	Random Forest	0.95 (0.89, 0.97)	0.95	0.94	0.94
		Support Vector Machine	0.93 (0.84, 0.96)	0.95	0.90	0.93

1 The values under the brackets correspond to 95% confidence interval (95% CI).

2 AUC represents the area under curve.

**Table 3.** Subgroup analysis of gene signatures expression and patient prognosis according to serous ovarian cancer grade treated with platinum-paclitaxel.

Grade	Genes	Hazard Ratio	Median Survival (Months) Low/High Expression	*p*-Value

Overall Survival (Grades 1 and 2)	ICAM1	0.55 (0.31–0.97)	48/66.57	*p* = 0.035
	TUBB2A	1.96 (1.17–3.29)	61.53/52	*p* < 0.01
	GLDC	1.91 (1.21–3.04)	66.57/48	*p* < 0.01
	PLAU	1.89 (1.13–3.18)	57/48	*p* = 0.014
	AURKA	1.74 (1.1–2.76)	61.53/44.3	*p* = 0.016
	NEAT1	1.62 (0.93–2.83)	83/52.63	*p* = 0.086
	MXRA5	1.71 (1.08–2.72)	61.53/52.63	*p* = 0.022
	GSN	0.82 (0.52–1.3)	52.63/57	*p* = 0.4
	MUC16	1.38 (0.87–2.19)	58/54.67	*p* = 0.17

Overall Survival (Grade 3)	ICAM1	1.12 (0.86–1.45)	42.13/41.83	*p* = 0.39
	TUBB2A	1.42(1.12–1.8)	45.77/38.47	*p* < 0.01
	GLDC	1.34 (1.04–1.74)	45.23/41.6	*p* = 0.025
	PLAU	0.78 (0.6–1.02)	36.77/45.53	*p* = 0.065
	AURKA	1.41 (1.1–1.81)	48.37/38.73	*p* < 0.01
	NEAT1	1.24 (0.98–1.58)	45.63/38.47	*p* = 0.073
	MXRA5	1.19 (0.92–1.54)	42.6/41.83	*p* = 0.19
	GSN	0.82 (0.63–1.08)	41/45.23	*p* = 0.17
	MUC16	0.89 (0.7–1.15)	38.4/44.8	*p* = 0.38

**Table 4.** Subgroup analysis of gene signatures expression and patient prognosis according to serous ovarian cancer treated with platinum-only.

Grade	Genes	Hazard Ratio	Median Survival (Months) Low/High Expression	p-Value

Overall Survival (Grade 1 + 2)	FCGBP	1.23 (0.86–1.76)	56.27/52.63	*p* = 0.25
	HSPA2	1.54 (0.99–2.4)	61.53/52.63	*p* = 0.05
	TFPI	1.28 (0.89–1.83)	57.1/48.27	*p* = 0.18
	NUAK1	1.92 (1.33–2.76)	60.5/37.13	*p* < 0.01
	LRRC17	1.84 (1.28–2.64)	62.47/45.4	*p* < 0.01
	FLRT2	1.66 (1.15–2.39)	59.33/48	*p* < 0.01
	FOXM1	0.77(0.54–1.1)	48.27/57.33	*p* = 0.14
	MAP4K2	1.46 (0.96–2.21)	65.17/52	*p* = 0.073
	CDC20	1.23 (0.86–1.75)	57/48.37	*p* = 0.26
	IL12A	1.27(0.89–1.83)	57.1/50	*p* = 0.18

Overall Survival (Grade 3)	FCGBP	1.47(1.2–1.79)	46/35.77	*p* < 0.01
	HSPA2	1.3 (1.08–1.58)	46/38.77	*p* < 0.01
	TFPI	1.16 (0.95–1.41)	45.77/41.57	*p* = 0.14
	NUAK1	1.39 (1.15–1.69)	45.77/40.07	*p* < 0.01
	LRRC17	1.3 (1.07–1.57)	45.77/38.43	*p* < 0.01
	FLRT2	1.26 (1.04–1.53)	45.77/40.1	*p* = 0.02
	FOXM1	0.81 (0.65–1.01)	42/48	*p* = 0.058
	MAP4K2	1.16 (0.95–1.4)	45.53/41.57	*p* = 0.14
	CDC20	0.85 (0.69–1.05)	38.4/45.47	*p* = 0.13
	IL12A	0.86 (0.7–1.05)	38.57/45.63	*p* = 0.14

**Table 5.** Efficacy prediction for alternative treatment options.

	Platinum-Paclitaxel Responder	Platinum-Paclitaxel Non-Responder

Responder with Platinum model	25 (26.88%)	34 (36.55%)
Non-responder with Platinum model	16 (17.20%)	18 (19.35%)

	Platinum Responder	Platinum Non-Responder

Responder with Platinum-Paclitaxel model	0 (0.0%)	0 (0.0%)
Non-responder with Platinum-Paclitaxel model	31 (38.75%)	49 (61.25%)

## Data Availability

The datasets presented in this study can be found in online repositories. The names of the repository/repositories and accession number(s) can be found in the article.

## References

[1] Sung H; Ferlay J; Siegel RL; Laversanne M; Soerjomataram I; Jemal A; Bray F Global Cancer Statistics 2020: GLOBOCAN Estimates of Incidence and Mortality Worldwide for 36 Cancers in 185 Countries. CA Cancer J. Clin. 2021, 71, 209–249. 33538338 10.3322/caac.21660

[2] Siegel RL; Miller KD; Fuchs HE; Jemal A Cancer statistics, 2022. CA Cancer J. Clin. 2022, 72, 7–33. 35020204 10.3322/caac.21708

[3] Prat J New insights into ovarian cancer pathology. Ann. Oncol. 2012, 23, x111–x117. 22987944 10.1093/annonc/mds300

[4] Garzon S; Laganà AS; Casarin J; Raffaelli R; Cromi A; Franchi M; Barra F; Alkatout I; Ferrero S; Ghezzi F Secondary and tertiary ovarian cancer recurrence: What is the best management? Gland. Surg. 2020, 9, 1118–1129. 32953627 10.21037/gs-20-325 PMC7475365

[5] McCluggage WG Morphological subtypes of ovarian carcinoma: A review with emphasis on new developments and pathogenesis. Pathology 2011, 43, 420–432. 21716157 10.1097/PAT.0b013e328348a6e7

[6] Guadagno E; Pignatiello S; Borrelli G; Cervasio M; Della Corte L; Bifulco G; Insabato L Ovarian borderline tumors, a subtype of neoplasm with controversial behavior. Role of Ki67 as a prognostic factor. Pathol.-Res. Pract. 2019, 215, 152633. 31542184 10.1016/j.prp.2019.152633

[7] Reade CJ; McVey RM; Tone AA; Finlayson SJ; McAlpine JN; Fung-Kee-Fung M; Ferguson SE The fallopian tube as the origin of high grade serous ovarian cancer: Review of a paradigm shift. J. Obstet. Gynaecol. Can. 2014, 36, 133–140. 24518912 10.1016/S1701-2163(15)30659-9

[8] Atallah GA; Kampan NC; Chew KT; Mohd Mokhtar N; Md Zin RR; Shafiee M.N.b.; Aziz N.H.b.A. Predicting Prognosis and Platinum Resistance in Ovarian Cancer: Role of Immunohistochemistry Biomarkers. Int. J. Mol. Sci. 2023, 24, 1973. 36768291 10.3390/ijms24031973 PMC9916805

[9] Vang R; Shih I-M; Kurman RJ Ovarian low-grade and high-grade serous carcinoma: Pathogenesis, clinicopathologic and molecular biologic features, and diagnostic problems. Adv. Anat. Pathol. 2009, 16, 267. 19700937 10.1097/PAP.0b013e3181b4fffa PMC2745605

[10] Torre LA; Trabert B; DeSantis CE; Miller KD; Samimi G; Runowicz CD; Gaudet MM; Jemal A; Siegel RL Ovarian cancer statistics, 2018. CA Cancer J. Clin. 2018, 68, 284–296. 29809280 10.3322/caac.21456 PMC6621554

[11] Wang EW; Wei CH; Liu S; Lee SJ-J; Shehayeb S; Glaser S; Li R; Saadat S; Shen J; Dellinger T Frontline Management of Epithelial Ovarian Cancer—Combining Clinical Expertise with Community Practice Collaboration and Cutting-Edge Research. J. Clin. Med. 2020, 9, 2830. 32882942 10.3390/jcm9092830 PMC7565288

[12] Cannistra SA Cancer of the ovary. N. Engl. J. Med. 2004, 351, 2519–2529. 15590954 10.1056/NEJMra041842

[13] Friedlander M; Matulonis U; Gourley C; du Bois A; Vergote I; Rustin G; Scott C; Meier W; Shapira-Frommer R; Safra T; Long-term efficacy, tolerability and overall survival in patients with platinum-sensitive, recurrent high-grade serous ovarian cancer treated with maintenance olaparib capsules following response to chemotherapy. Br. J. Cancer 2018, 119, 1075–1085. 30353045 10.1038/s41416-018-0271-y PMC6219499

[14] Therasse P; Arbuck SG; Eisenhauer EA; Wanders J; Kaplan RS; Rubinstein L; Verweij J; Van Glabbeke M; van Oosterom AT; Christian MC; New guidelines to evaluate the response to treatment in solid tumors. European Organization for Research and Treatment of Cancer, National Cancer Institute of the United States, National Cancer Institute of Canada. J. Natl. Cancer Inst. 2000, 92, 205–216. 10655437 10.1093/jnci/92.3.205

[15] Friedlander M; Butow P; Stockler M; Gainford C; Martyn J; Oza A; Donovan HS; Miller B; King M Symptom control in patients with recurrent ovarian cancer: Measuring the benefit of palliative chemotherapy in women with platinum refractory/resistant ovarian cancer. Int. J. Gynecol. Cancer 2009, 19 (Suppl. 2), S44–S48. 19955914 10.1111/IGC.0b013e3181bf7fb8

[16] Gonzalez Bosquet J; Devor EJ; Newtson AM; Smith BJ; Bender DP; Goodheart MJ; McDonald ME; Braun TA; Thiel KW; Leslie KK Creation and validation of models to predict response to primary treatment in serous ovarian cancer. Sci. Rep. 2021, 11, 5957. 33727600 10.1038/s41598-021-85256-9 PMC7971042

[17] Walker JL; Brady MF; Wenzel L; Fleming GF; Huang HQ; DiSilvestro PA; Fujiwara K; Alberts DS; Zheng W; Tewari KS; Randomized Trial of Intravenous Versus Intraperitoneal Chemotherapy Plus Bevacizumab in Advanced Ovarian Carcinoma: An NRG Oncology/Gynecologic Oncology Group Study. J. Clin. Oncol. 2019, 37, 1380–1390. 31002578 10.1200/JCO.18.01568 PMC6544459

[18] Baekelandt M; Kristensen GB; Nesland JM; Tropé CG; Holm R Clinical significance of apoptosis-related factors p53, Mdm2, and Bcl-2 in advanced ovarian cancer. J. Clin. Oncol. 1999, 17, 2061. 10561259 10.1200/JCO.1999.17.7.2061

[19] Baekelandt M; Holm R; Nesland JM; Tropé CG; Kristensen GB Expression of apoptosis-related proteins is an independent determinant of patient prognosis in advanced ovarian cancer. J. Clin. Oncol. 2000, 18, 3775–3781. 11078490 10.1200/JCO.2000.18.22.3775

[20] Abu Samaan TM; Samec M; Liskova A; Kubatka P; Büsselberg D Paclitaxel’s Mechanistic and Clinical Effects on Breast Cancer. Biomolecules 2019, 9, 789. 31783552 10.3390/biom9120789 PMC6995578

[21] Weaver BA How Taxol/paclitaxel kills cancer cells. Mol. Biol. Cell 2014, 25, 2677–2681. 25213191 10.1091/mbc.E14-04-0916 PMC4161504

[22] Nezi L; Musacchio A Sister chromatid tension and the spindle assembly checkpoint. Curr. Opin. Cell Biol. 2009, 21, 785–795. 19846287 10.1016/j.ceb.2009.09.007

[23] Lu T-P; Kuo K-T; Chen C-H; Chang M-C; Lin H-P; Hu Y-H; Chiang Y-C; Cheng W-F; Chen C-A Developing a Prognostic Gene Panel of Epithelial Ovarian Cancer Patients by a Machine Learning Model. Cancers 2019, 11, 270. 30823599 10.3390/cancers11020270 PMC6406249

[24] Yu K-H; Levine DA; Zhang H; Chan DW; Zhang Z; Snyder M Predicting Ovarian Cancer Patients’ Clinical Response to Platinum-Based Chemotherapy by Their Tumor Proteomic Signatures. J. Proteome Res. 2016, 15, 2455–2465. 27312948 10.1021/acs.jproteome.5b01129 PMC8718213

[25] Amniouel S; Jafri MS High-accuracy prediction of colorectal cancer chemotherapy efficacy using machine learning applied to gene expression data. Front. Physiol. 2024, 14, 1272206. 38304289 10.3389/fphys.2023.1272206 PMC10830836

[26] Gharaibeh RZ; Fodor AA; Gibas CJ Background correction using dinucleotide affinities improves the performance of GCRMA. BMC Bioinform. 2008, 9, 452. 10.1186/1471-2105-9-452 PMC2579310 18947404

[27] Irizarry RA; Bolstad BM; Collin F; Cope LM; Hobbs B; Speed TP Summaries of Affymetrix GeneChip probe level data. Nucleic Acids Res. 2003, 31, e15. 12582260 10.1093/nar/gng015 PMC150247

[28] Irizarry RA; Warren D; Spencer F; Kim IF; Biswal S; Frank BC; Gabrielson E; Garcia JG; Geoghegan J; Germino G; Multiple-laboratory comparison of microarray platforms. Nat. Methods 2005, 2, 345–350. 15846361 10.1038/nmeth756

[29] Tilford CA; Siemers NO Gene set enrichment analysis. Methods Mol. Biol. 2009, 563, 99–121. 19597782 10.1007/978-1-60761-175-2_6

[30] Kauffmann A; Gentleman R; Huber W arrayQualityMetrics—A bioconductor package for quality assessment of microarray data. Bioinformatics 2009, 25, 415–416. 19106121 10.1093/bioinformatics/btn647 PMC2639074

[31] Kauffmann A; Huber W Microarray data quality control improves the detection of differentially expressed genes. Genomics 2010, 95, 138–142. 20079422 10.1016/j.ygeno.2010.01.003

[32] Tweedie S; Braschi B; Gray K; Jones TE; Seal RL; Yates B; Bruford EA Genenames. org: The HGNC and VGNC resources in 2021. Nucleic Acids Res. 2021, 49, D939–D946. 33152070 10.1093/nar/gkaa980 PMC7779007

[33] Braschi B; Seal RL; Tweedie S; Jones TE; Bruford EA The risks of using unapproved gene symbols. Am. J. Hum. Genet. 2021, 108, 1813–1816. 34626580 10.1016/j.ajhg.2021.09.004 PMC8546045

[34] Carlson MR; Pagès H; Arora S; Obenchain V; Morgan M Genomic Annotation Resources in R/Bioconductor. Methods Mol. Biol. 2016, 1418, 67–90. 27008010 10.1007/978-1-4939-3578-9_4

[35] Cheadle C; Vawter MP; Freed WJ; Becker KG Analysis of microarray data using Z score transformation. J. Mol. Diagn. 2003, 5, 73–81. 12707371 10.1016/S1525-1578(10)60455-2 PMC1907322

[36] Yasrebi H Comparative study of joint analysis of microarray gene expression data in survival prediction and risk assessment of breast cancer patients. Brief. Bioinform. 2016, 17, 771–785. 26504096 10.1093/bib/bbv092 PMC5863785

[37] Leek JT; Johnson WE; Parker HS; Jaffe AE; Storey JD The sva package for removing batch effects and other unwanted variation in high-throughput experiments. Bioinformatics 2012, 28, 882–883. 22257669 10.1093/bioinformatics/bts034 PMC3307112

[38] Jung Y; Hu J A K-fold Averaging Cross-validation Procedure. J. Nonparametr. Stat. 2015, 27, 167–179. 27630515 10.1080/10485252.2015.1010532 PMC5019184

[39] Kairalla JA; Coffey CS; Muller KE GLUMIP 2.0: SAS/IML Software for Planning Internal Pilots. J. Stat. Softw. 2008, 28, 1–32. 27774042 10.18637/jss.v028.i07 PMC5074077

[40] Ritchie ME; Phipson B; Wu D; Hu Y; Law CW; Shi W; Smyth GK limma powers differential expression analyses for RNA-sequencing and microarray studies. Nucleic Acids Res. 2015, 43, e47. 25605792 10.1093/nar/gkv007 PMC4402510

[41] Friedman J; Hastie T; Tibshirani R Regularization paths for generalized linear models via coordinate descent. J. Stat. Softw. 2010, 33, 1. 20808728 PMC2929880

[42] Ghosh Roy G; Geard N; Verspoor K; He S PoLoBag: Polynomial Lasso Bagging for signed gene regulatory network inference from expression data. Bioinformatics 2021, 36, 5187–5193. 32697830 10.1093/bioinformatics/btaa651

[43] Hua J LAK: Lasso and K-Means Based Single-Cell RNA-Seq Data Clustering Analysis. IEEE Access 2020, 8, 129679–129688.

[44] Diaz-Uriarte R GeneSrF and varSelRF: A web-based tool and R package for gene selection and classification using random forest. BMC Bioinform. 2007, 8, 328. 10.1186/1471-2105-8-328 PMC2034606 17767709

[45] Rigatti SJ Random Forest. J. Insur. Med. 2017, 47, 31–39. 28836909 10.17849/insm-47-01-31-39.1

[46] Meyer D Support Vector Machines The Interface to libsvm in package e1071. R. News 2001, 1, 23–26.

[47] He Z; Liu Z; Gong L Biomarker identification and pathway analysis of rheumatoid arthritis based on metabolomics in combination with ingenuity pathway analysis. Proteomics 2021, 21, 2100037. 10.1002/pmic.202100037 33969925

[48] Ferriss JS; Kim Y; Duska L; Birrer M; Levine DA; Moskaluk C; Theodorescu D; Lee JK Multi-Gene Expression Predictors of Single Drug Responses to Adjuvant Chemotherapy in Ovarian Carcinoma: Predicting Platinum Resistance. PLoS ONE 2012, 7, e30550. 22348014 10.1371/journal.pone.0030550 PMC3277593

[49] Ortiz M; Wabel E; Mitchell K; Horibata S Mechanisms of chemotherapy resistance in ovarian cancer. Cancer Drug Resist. 2022, 5, 304–316. 35800369 10.20517/cdr.2021.147 PMC9255249

[50] Zhou J; Kang Y; Chen L; Wang H; Liu J; Zeng S; Yu L The Drug-Resistance Mechanisms of Five Platinum-Based Antitumor Agents. Front. Pharmacol. 2020, 11, 343. 32265714 10.3389/fphar.2020.00343 PMC7100275

[51] Mondal P; Meeran SM Emerging role of non-coding RNAs in resistance to platinum-based anti-cancer agents in lung cancer. Front. Pharmacol. 2023, 14, 1105484. 36778005 10.3389/fphar.2023.1105484 PMC9909610

[52] Basu A; Krishnamurthy S Cellular Responses to Cisplatin-Induced DNA Damage. J. Nucleic Acids 2010, 2010, 201367. 20811617 10.4061/2010/201367 PMC2929606

[53] Sazonova EV; Kopeina GS; Imyanitov EN; Zhivotovsky B Platinum drugs and taxanes: Can we overcome resistance? Cell Death Discov. 2021, 7, 155. 34226520 10.1038/s41420-021-00554-5 PMC8257727

[54] Cummings M; Freer C; Orsi NM Targeting the tumour microenvironment in platinum-resistant ovarian cancer. Semin. Cancer Biol. 2021, 77, 3–28. 33607246 10.1016/j.semcancer.2021.02.007

[55] Londero AP; Orsaria M; Tell G; Marzinotto S; Capodicasa V; Poletto M; Vascotto C; Sacco C; Mariuzzi L Expression and Prognostic Significance of APE1/Ref-1 and NPM1 Proteins in High-Grade Ovarian Serous Cancer. Am. J. Clin. Pathol. 2014, 141, 404–414. 24515769 10.1309/AJCPIDKDLSGE26CX

[56] Wike CL; Graves HK; Hawkins R; Gibson MD; Ferdinand MB; Zhang T; Chen Z; Hudson DF; Ottesen JJ; Poirier MG; Aurora-A mediated histone H3 phosphorylation of threonine 118 controls condensin I and cohesin occupancy in mitosis. Elife 2016, 5, e11402. 26878753 10.7554/eLife.11402 PMC4798946

[57] Coughlan AY; Testa G Exploiting epigenetic dependencies in ovarian cancer therapy. Int. J. Cancer 2021, 149, 1732–1743. 34213777 10.1002/ijc.33727 PMC9292863

[58] Yang C; Zhang J; Ma Y; Wu C; Cui W; Wang L Histone methyltransferase and drug resistance in cancers. J. Exp. Clin. Cancer Res. 2020, 39, 173. 32859239 10.1186/s13046-020-01682-z PMC7455899

[59] Wang S; Yin C; Zhang Y; Zhang L; Tao L; Liang W; Pang L; Fu R; Ding Y; Li F; Overexpression of ICAM-1 Predicts Poor Survival in High-Grade Serous Ovarian Carcinoma: A Study Based on TCGA and GEO Databases and Tissue Microarray. Biomed. Res. Int. 2019, 2019, 2867372. 31312656 10.1155/2019/2867372 PMC6595389

[60] Zhan SJ; Liu B; Linghu H Identifying genes as potential prognostic indicators in patients with serous ovarian cancer resistant to carboplatin using integrated bioinformatics analysis. Oncol. Rep. 2018, 39, 2653–2663. 29693178 10.3892/or.2018.6383 PMC5983937

[61] Katsha A; Belkhiri A; Goff L; El-Rifai W Aurora kinase A in gastrointestinal cancers: Time to target. Mol. Cancer 2015, 14, 106. 25987188 10.1186/s12943-015-0375-4 PMC4436812

[62] Du R; Huang C; Liu K; Li X; Dong Z Targeting AURKA in Cancer: Molecular mechanisms and opportunities for Cancer therapy. Mol. Cancer 2021, 20, 15. 33451333 10.1186/s12943-020-01305-3 PMC7809767

[63] Buckanovich RJ; Sasaroli D; O’Brien-Jenkins A; Botbyl J; Hammond R; Katsaros D; Sandaltzopoulos R; Liotta LA; Gimotty PA; Coukos G Tumor vascular proteins as biomarkers in ovarian cancer. J. Clin. Oncol. 2007, 25, 852–861. 17327606 10.1200/JCO.2006.08.8583

[64] Peng S.-q.; Zhu X.-r.; Zhao M.-z.; Zhang Y.-f.; Wang A.-r.; Chen M.-b.; Ye Z.-y. Identification of matrix-remodeling associated 5 as a possible molecular oncotarget of pancreatic cancer. Cell Death Dis. 2023, 14, 157. 36828810 10.1038/s41419-023-05684-5 PMC9958022

[65] Minafra L; Bravatà V; Forte GI; Cammarata FP; Gilardi MC; Messa C Gene expression profiling of epithelial-mesenchymal transition in primary breast cancer cell culture. Anticancer Res. 2014, 34, 2173–2183. 24778019

[66] Yin L; Wang Y Long non-coding RNA NEAT1 facilitates the growth, migration, and invasion of ovarian cancer cells via the let-7 g/MEST/ATGL axis. Cancer Cell Int. 2021, 21, 437. 34416900 10.1186/s12935-021-02018-3 PMC8379830

[67] Yong W; Yu D; Jun Z; Yachen D; Weiwei W; Midie X; Xingzhu J; Xiaohua W Long noncoding RNA NEAT1, regulated by LIN28B, promotes cell proliferation and migration through sponging miR-506 in high-grade serous ovarian cancer. Cell Death Dis. 2018, 9, 861. 30154460 10.1038/s41419-018-0908-z PMC6113267

[68] Chen ZJ; Zhang Z; Xie BB; Zhang HY Clinical significance of up-regulated lncRNA NEAT1 in prognosis of ovarian cancer. Eur. Rev. Med. Pharmacol. Sci. 2016, 20, 3373–3377. 27608895

[69] Knutsen E; Harris AL; Perander M Expression and functions of long non-coding RNA NEAT1 and isoforms in breast cancer. Br. J. Cancer 2022, 126, 551–561. 34671127 10.1038/s41416-021-01588-3 PMC8854383

[70] Wang X; Shi J; Huang M; Chen J; Dan J; Tang Y; Guo Z; He X; Zhao Q TUBB2B facilitates progression of hepatocellular carcinoma by regulating cholesterol metabolism through targeting HNF4A/CYP27A1. Cell Death Dis. 2023, 14, 179. 36872411 10.1038/s41419-023-05687-2 PMC9986231

[71] Kwon H; Oh S; Jin X; An YJ; Park S Cancer metabolomics in basic science perspective. Arch. Pharmacal Res. 2015, 38, 372–380. 10.1007/s12272-015-0552-4 25630795

[72] Shin S-J; Chung H; Kim JY; Kim H; Cho C-H; Ha E Abstract 5748: Downregulation of glycine decarboxylase renders ovarian cancer cells less proliferative and more chemoresistant. Cancer Res. 2018, 78, 5748.

[73] Fang Z; Xu S; Xie Y; Yan W Identification of a prognostic gene signature of colon cancer using integrated bioinformatics analysis. World J. Surg. Oncol. 2021, 19, 13. 33441161 10.1186/s12957-020-02116-y PMC7807455

[74] Li J; Fan H; Zhou X; Xiang Y; Liu Y Prognostic Significance and Gene Co-Expression Network of PLAU and PLAUR in Gliomas. Front. Oncol. 2022, 11, 602321. 35087738 10.3389/fonc.2021.602321 PMC8787124

[75] Zhai Y; Lu Q; Lou T; Cao G; Wang S; Zhang Z MUC16 affects the biological functions of ovarian cancer cells and induces an antitumor immune response by activating dendritic cells. Ann. Transl. Med. 2020, 8, 1494. 33313239 10.21037/atm-20-6388 PMC7729312

[76] Felder M; Kapur A; Gonzalez-Bosquet J; Horibata S; Heintz J; Albrecht R; Fass L; Kaur J; Hu K; Shojaei H; MUC16 (CA125): Tumor biomarker to cancer therapy, a work in progress. Mol. Cancer 2014, 13, 129. 24886523 10.1186/1476-4598-13-129 PMC4046138

[77] Lakshmanan I; Ponnusamy MP; Das S; Chakraborty S; Haridas D; Mukhopadhyay P; Lele SM; Batra SK MUC16 induced rapid G2/M transition via interactions with JAK2 for increased proliferation and anti-apoptosis in breast cancer cells. Oncogene 2012, 31, 805–817. 21785467 10.1038/onc.2011.297 PMC3288594

[78] Abedini MR; Wang P-W; Huang Y-F; Cao M; Chou C-Y; Shieh D-B; Tsang BK Cell fate regulation by gelsolin in human gynecologic cancers. Proc. Natl. Acad. Sci. USA 2014, 111, 14442–14447. 25246592 10.1073/pnas.1401166111 PMC4209992

[79] Arentz G; Mittal P; Klingler-Hoffmann M; Condina MR; Ricciardelli C; Lokman NA; Kaur G; Oehler MK; Hoffmann P Label-Free Quantification Mass Spectrometry Identifies Protein Markers of Chemotherapy Response in High-Grade Serous Ovarian Cancer. Cancers 2023, 15, 2172. 37046833 10.3390/cancers15072172 PMC10093294

[80] Kim SI; Hwangbo S; Dan K; Kim HS; Chung HH; Kim J-W; Park NH; Song Y-S; Han D; Lee M Proteomic Discovery of Plasma Protein Biomarkers and Development of Models Predicting Prognosis of High-Grade Serous Ovarian Carcinoma. Mol. Cell. Proteom. 2023, 22, 100502. 10.1016/j.mcpro.2023.100502 PMC9972571 36669591

[81] Onuma T; Asare-Werehene M; Yoshida Y; Tsang BK Exosomal Plasma Gelsolin Is an Immunosuppressive Mediator in the Ovarian Tumor Microenvironment and a Determinant of Chemoresistance. Cells 2022, 11, 3305. 36291171 10.3390/cells11203305 PMC9600545

[82] Zhang W; Wang Y Activation of RIPK2-mediated NOD1 signaling promotes proliferation and invasion of ovarian cancer cells via NF-κB pathway. Histochem. Cell Biol. 2022, 157, 173–182. 34825931 10.1007/s00418-021-02055-z

[83] Velloso FJ; Campos AR; Sogayar MC; Correa RG Proteome profiling of triple negative breast cancer cells overexpressing NOD1 and NOD2 receptors unveils molecular signatures of malignant cell proliferation. BMC Genom. 2019, 20, 152. 10.1186/s12864-019-5523-6 PMC6385390 30791886

[84] Abedini MR; Muller EJ; Bergeron R; Gray DA; Tsang BK Akt promotes chemoresistance in human ovarian cancer cells by modulating cisplatin-induced, p53-dependent ubiquitination of FLICE-like inhibitory protein. Oncogene 2010, 29, 11–25. 19802016 10.1038/onc.2009.300

[85] Abedini MR; Muller EJ; Brun J; Bergeron R; Gray DA; Tsang BK Cisplatin Induces p53-Dependent FLICE-Like Inhibitory Protein Ubiquitination in Ovarian Cancer Cells. Cancer Res. 2008, 68, 4511–4517. 18559494 10.1158/0008-5472.CAN-08-0673

[86] Phippen NT; Bateman NW; Wang G; Hamilton CA; Maxwell GL; Darcy KM; Conrads TP Abstract 4632: Poor survival associated with NUAK1 overexpression in serous ovarian cancer may be explained by chemotherapy resistance. Cancer Res. 2015, 75, 4632.

[87] Hou X; Liu JE; Liu W; Liu CY; Liu ZY; Sun ZY A new role of NUAK1: Directly phosphorylating p53 and regulating cell proliferation. Oncogene 2011, 30, 2933–2942. 21317932 10.1038/onc.2011.19

[88] Oh C-K; Park JJ; Ha M; Heo HJ; Kang J; Kwon EJ; Kang JW; Kim Y; Kang JM; Yoon SZ; *LRRC17* Is Linked to Prognosis of Ovarian Cancer Through a p53-dependent Anti-apoptotic Function. Anticancer Res. 2020, 40, 5601–5609. 32988884 10.21873/anticanres.14573

[89] Kidokoro T; Tanikawa C; Furukawa Y; Katagiri T; Nakamura Y; Matsuda K CDC20, a potential cancer therapeutic target, is negatively regulated by p53. Oncogene 2008, 27, 1562–1571. 17873905 10.1038/sj.onc.1210799

[90] Xi X; Cao T; Qian Y; Wang H; Ju S; Chen Y; Chen T; Yang J; Liang B; Hou S CDC20 is a novel biomarker for improved clinical predictions in epithelial ovarian cancer. Am. J. Cancer Res. 2022, 12, 3303–3317. 35968331 PMC9360218

[91] Liu C; Barger CJ; Karpf AR FOXM1: A Multifunctional Oncoprotein and Emerging Therapeutic Target in Ovarian Cancer. Cancers 2021, 13, 3065. 34205406 10.3390/cancers13123065 PMC8235333

[92] Guo X; Song C; Fang L; Li M; Yue L; Sun Q FLRT2 functions as Tumor Suppressor gene inactivated by promoter methylation in Colorectal Cancer. J. Cancer 2020, 11, 7329–7338. 33193897 10.7150/jca.47558 PMC7646184

[93] Vivier E; Nunès JA; Vély F Natural killer cell signaling pathways. Science 2004, 306, 1517–1519. 15567854 10.1126/science.1103478

[94] Gonzalez VD; Huang Y-W; Delgado-Gonzalez A; Chen S-Y; Donoso K; Sachs K; Gentles AJ; Allard GM; Kolahi KS; Howitt BE; High-grade serous ovarian tumor cells modulate NK cell function to create an immune-tolerant microenvironment. Cell Rep. 2021, 36, 109632. 34469729 10.1016/j.celrep.2021.109632 PMC8546503

[95] Parihar R; Dierksheide J; Hu Y; Carson WE IL-12 enhances the natural killer cell cytokine response to Ab-coated tumor cells. J. Clin. Investig. 2002, 110, 983–992. 12370276 10.1172/JCI15950 PMC151155

[96] Rao Z; Ding Y Ubiquitin pathway and ovarian cancer. Curr. Oncol. 2012, 19, 324–328. 23300358 10.3747/co.19.1175 PMC3503665

[97] Sojka DR; Abramowicz A; Adamiec-Organiściok M; Karnas E; Mielańczyk Ł; Kania D; Blamek S; Telka E; Scieglinska D Heat shock protein A2 is a novel extracellular vesicle-associated protein. Sci. Rep. 2023, 13, 4734. 36959387 10.1038/s41598-023-31962-5 PMC10036471

[98] Hoter A; Naim HY Heat Shock Proteins and Ovarian Cancer: Important Roles and Therapeutic Opportunities. Cancers 2019, 11, 1389. 31540420 10.3390/cancers11091389 PMC6769485

[99] Wang Y; Liu Y; Liu H; Zhang Q; Song H; Tang J; Fu J; Wang X FcGBP was upregulated by HPV infection and correlated to longer survival time of HNSCC patients. Oncotarget 2017, 8, 86503–86514. 29156811 10.18632/oncotarget.21220 PMC5689701

[100] Koizume S; Miyagi Y Potential Coagulation Factor-Driven Pro-Inflammatory Responses in Ovarian Cancer Tissues Associated with Insufficient O2 and Plasma Supply. Int. J. Mol. Sci. 2017, 18, 809. 28417928 10.3390/ijms18040809 PMC5412393

[101] Koizume S; Miyagi Y Tissue Factor–Factor VII Complex as a Key Regulator of Ovarian Cancer Phenotypes. Biomark. Cancer 2015, 7, BIC-S29318. 10.4137/BIC.S29318 PMC4562604 26396550

[102] Miyake R; Yamada Y; Yamanaka S; Kawaguchi R; Ootake N; Myoba S; Kobayashi H Tissue factor pathway inhibitor 2 as a serum marker for diagnosing sasymptomatic venous thromboembolism in patients with epithelial ovarian cancer and positive D-dimer results. Mol. Clin. Oncol. 2022, 16, 46. 35003744 10.3892/mco.2021.2479 PMC8739701

[103] Wang X; Wang E; Kavanagh JJ; Freedman RS Ovarian cancer, the coagulation pathway, and inflammation. J. Transl. Med. 2005, 3, 25. 15969748 10.1186/1479-5876-3-25 PMC1182397

[104] Judson PL; Watson JM; Gehrig PA; Fowler WC Jr.; Haskill JS Cisplatin Inhibits Paclitaxel-induced Apoptosis in Cisplatin-resistant Ovarian Cancer Cell Lines: Possible Explanation for Failure ofCombination Therapy1. Cancer Res. 1999, 59, 2425–2432. 10344753

[105] Choi HS; Kim Y-K; Hwang K-G; Yun P-Y Increased FOXM1 Expression by Cisplatin Inhibits Paclitaxel-Related Apoptosis in Cisplatin-Resistant Human Oral Squamous Cell Carcinoma (OSCC) Cell Lines. Int. J. Mol. Sci. 2020, 21, 8897. 33255409 10.3390/ijms21238897 PMC7727786

[106] Fonti V; Belitser E Feature selection using lasso. VU Amst. Res. Pap. Bus. Anal. 2017, 30, 1–25.

[107] Speiser JL; Miller ME; Tooze J; Ip E A comparison of random forest variable selection methods for classification prediction modeling. Expert Syst. Appl. 2019, 134, 93–101. 32968335 10.1016/j.eswa.2019.05.028 PMC7508310

