# Supplementary material for: Evaluating Ovarian Cancer Chemotherapy Response Using Gene Expression Data and Machine Learning
Source: BioMedInformatics. Author manuscript; Available in PMC 2024 Aug 15. (PMC11326537; doi:10.3390/biomedinformatics4020077)
Supplement: Supplement [file NIHMS2013972-supplement-Supplement.docx]

**Table S1:** Differentially expressed genes identified between responders and non-responders of platinum-paclitaxel in patients with serous ovarian cancer.

| **Gene.symbol** | **logFC** | **AveExpr** | **t** | **P.Value** | **log2FoldChange** | **P.Adjusted Value** |
| --- | --- | --- | --- | --- | --- | --- |
| ICAM1 | 1.258712 | 4.712829 | 3.825611 | 0.000355 | 2.39282 | 0.021741 |
| NEAT1 | 1.750267 | 7.9721 | 3.660446 | 0.000594 | 3.364209 | 0.021741 |
| MUC4 | 1.870482 | 8.740564 | 3.585137 | 0.000749 | 3.656548 | 0.021741 |
| CAPZB | 1.102599 | 4.822414 | 3.564364 | 0.000798 | 2.147411 | 0.021741 |
| THSD4 | 1.945427 | 6.571429 | 3.486054 | 0.001012 | 3.851518 | 0.022059 |
| LOC105370109 | 1.325439 | 4.488578 | 3.299423 | 0.001764 | 2.506091 | 0.029612 |
| MXRA5 | 1.303457 | 4.711662 | 3.233476 | 0.002138 | 2.468197 | 0.029612 |
| CALR | 1.06549 | 6.106294 | 3.169809 | 0.002569 | 2.092881 | 0.029612 |
| RDH13 | 1.409014 | 4.5503 | 3.126192 | 0.002911 | 2.655556 | 0.029612 |
| MUC16 | 1.259785 | 4.347569 | 3.120342 | 0.00296 | 2.3946 | 0.029612 |
| PLAU | 1.059728 | 3.07657 | 3.116989 | 0.002988 | 2.084539 | 0.029612 |
| TUBB2A | 1.510156 | 6.142812 | 3.073643 | 0.003379 | 2.848408 | 0.030695 |
| DCHS1 | 1.119577 | 5.361855 | 3.015963 | 0.003974 | 2.172832 | 0.032199 |
| ITGB5 | 1.724056 | 7.110435 | 2.966262 | 0.004564 | 3.303639 | 0.032199 |
| NRBP2 | 1.095633 | 5.895585 | 2.957295 | 0.004678 | 2.137068 | 0.032199 |
| TBL1XR1 | 1.513537 | 7.706752 | 2.941258 | 0.00489 | 2.855091 | 0.032199 |
| GLDC | 1.085652 | 3.396137 | 2.888187 | 0.005656 | 2.122335 | 0.032199 |
| PAN3 | 1.179783 | 6.569144 | 2.885462 | 0.005699 | 2.265426 | 0.032199 |
| IFT80 | 1.226002 | 4.825622 | 2.884846 | 0.005708 | 2.339179 | 0.032199 |
| MRI1 | 1.081992 | 5.414245 | 2.85229 | 0.006236 | 2.116957 | 0.032199 |
| PARD6B | 1.04961 | 5.473988 | 2.835552 | 0.006525 | 2.06997 | 0.032199 |
| SRRM2 | 1.245625 | 4.85799 | 2.835388 | 0.006528 | 2.371212 | 0.032199 |
| SNX19 | 1.104696 | 4.528653 | 2.806314 | 0.00706 | 2.150536 | 0.032199 |
| TIPRL | 1.575885 | 6.295907 | 2.804731 | 0.00709 | 2.981183 | 0.032199 |
| TIMP3 | 1.265991 | 5.549762 | 2.780104 | 0.007573 | 2.404924 | 0.033018 |
| CDR1 | 1.656838 | 6.518186 | 2.747068 | 0.008269 | 3.153246 | 0.033257 |
| SFT2D2 | 1.244536 | 6.663114 | 2.729754 | 0.008657 | 2.369423 | 0.033257 |
| AEBP1 | 1.367908 | 8.949637 | 2.725802 | 0.008748 | 2.58096 | 0.033257 |
| SRSF11 | 1.015878 | 6.647739 | 2.721468 | 0.008848 | 2.022133 | 0.033257 |
| CD81 | 1.035816 | 7.181112 | 2.679819 | 0.009871 | 2.050273 | 0.033756 |
| RBPMS | 1.305834 | 6.510124 | 2.679614 | 0.009876 | 2.472266 | 0.033756 |
| HSPA5 | 1.101874 | 7.735995 | 2.667748 | 0.010187 | 2.146332 | 0.033756 |
| PGLS | 1.094452 | 10.30997 | 2.666511 | 0.01022 | 2.135319 | 0.033756 |
| PPIC | 1.231994 | 7.191868 | 2.623606 | 0.011423 | 2.348915 | 0.034372 |
| ITM2B | 1.072594 | 6.989541 | 2.617335 | 0.011609 | 2.103211 | 0.034372 |
| NNMT | 1.736113 | 8.017737 | 2.617328 | 0.01161 | 3.331364 | 0.034372 |
| GSN | -1.11553 | 5.396278 | -2.61539 | 0.011668 | -2.16675 | 0.034372 |
| SNX21 | 1.12754 | 4.674479 | 2.599682 | 0.012149 | 2.184858 | 0.034848 |
| ESD | 1.08948 | 5.665136 | 2.568969 | 0.013143 | 2.127973 | 0.036732 |
| LAMP1 | 1.407333 | 10.41783 | 2.542265 | 0.014066 | 2.652464 | 0.037877 |
| VCAN | 1.092599 | 4.240653 | 2.530426 | 0.014494 | 2.132578 | 0.037877 |
| WNT6 | 1.992857 | 6.644343 | 2.527673 | 0.014595 | 3.980244 | 0.037877 |
| COL11A1 | 1.039985 | 3.259803 | 2.514979 | 0.01507 | 2.056206 | 0.0382 |
| SPARC | 1.113733 | 7.319859 | 2.494076 | 0.015882 | 2.164049 | 0.038537 |
| CDC42BPA | 1.068818 | 6.924712 | 2.493389 | 0.01591 | 2.097713 | 0.038537 |
| NRP2 | 1.044649 | 6.783994 | 2.441491 | 0.018105 | 2.062864 | 0.0429 |
| CHI3L1 | 1.495498 | 8.68154 | 2.421308 | 0.01903 | 2.819614 | 0.043454 |
| LUC7L3 | 1.412171 | 8.337008 | 2.417485 | 0.019209 | 2.661374 | 0.043454 |
| COL5A2 | 1.122494 | 6.9421 | 2.405 | 0.019808 | 2.17723 | 0.043454 |
| ABCC10 | 1.017467 | 4.527186 | 2.399509 | 0.020076 | 2.024362 | 0.043454 |
| LENG8 | 1.211602 | 10.24423 | 2.388984 | 0.020599 | 2.315947 | 0.043454 |
| AURKA | 1.109982 | 6.450443 | 2.386393 | 0.02073 | 2.158429 | 0.043454 |
| MRPL57 | 1.152862 | 6.191982 | 2.37146 | 0.021498 | 2.223546 | 0.044214 |
| SLC34A2 | 1.457094 | 5.979735 | 2.353086 | 0.022478 | 2.745548 | 0.044216 |
| SPON1 | 1.422028 | 7.917827 | 2.341612 | 0.023111 | 2.67962 | 0.044216 |
| CP | 1.345019 | 6.907464 | 2.339052 | 0.023254 | 2.540336 | 0.044216 |
| ARHGAP33 | 1.044922 | 5.432901 | 2.320916 | 0.024291 | 2.063255 | 0.044216 |
| SIDT2 | 1.30597 | 8.080496 | 2.318244 | 0.024447 | 2.472499 | 0.044216 |
| ANXA2 | 1.038303 | 7.476907 | 2.315426 | 0.024613 | 2.05381 | 0.044216 |
| XAF1 | 1.048172 | 6.313692 | 2.313144 | 0.024748 | 2.067909 | 0.044216 |
| ADAMTS10 | -1.07193 | 5.088951 | -2.30798 | 0.025056 | -2.10225 | 0.044216 |
| COL3A1 | 1.475464 | 6.934609 | 2.306405 | 0.025151 | 2.78073 | 0.044216 |
| KLK7 | 1.154481 | 6.604088 | 2.288665 | 0.026238 | 2.226042 | 0.044433 |
| ZNF503 | 1.003374 | 7.024202 | 2.287067 | 0.026338 | 2.004683 | 0.044433 |
| PTX3 | 1.069121 | 3.331447 | 2.284545 | 0.026497 | 2.098155 | 0.044433 |
| AHNAK2 | 1.010325 | 6.487022 | 2.26623 | 0.027673 | 2.014364 | 0.045252 |
| USP4 | 1.112193 | 6.038708 | 2.26406 | 0.027816 | 2.161739 | 0.045252 |
| FN1 | 1.457346 | 6.029537 | 2.249265 | 0.028804 | 2.746026 | 0.046171 |
| ACACB | 1.045382 | 6.943146 | 2.20679 | 0.031817 | 2.063912 | 0.050039 |
| HPS3 | 1.07159 | 5.923856 | 2.20202 | 0.032173 | 2.101748 | 0.050039 |
| COX5A | 1.103853 | 8.719836 | 2.196418 | 0.032594 | 2.149279 | 0.050039 |

**Table S2:** Differential expressed genes identified between responders and non-responders of platinum-only in patients with serous ovarian cancer.

| **Gene.symbol** | **logFC** | **AveExpr** | **t** | **P.Value** | **log2FoldChange** | **P.Adjusted Value** |
| --- | --- | --- | --- | --- | --- | --- |
| GNG11 | 1.335782 | 8.696821 | 3.878534 | 0.000377 | 2.524123 | 0.015882 |
| HSPA2 | 1.658692 | 9.024278 | 3.786319 | 0.000496 | 3.157302 | 0.015882 |
| ARHGAP6 | 1.603253 | 6.274051 | 3.72114 | 0.000601 | 3.038277 | 0.015882 |
| NCAPH | -1.43792 | 6.025163 | -3.6898 | 0.000659 | -2.7093 | 0.015882 |
| EPS8 | 1.167717 | 9.87811 | 3.573949 | 0.000925 | 2.246558 | 0.015882 |
| LMNB1 | -1.1388 | 8.587725 | -3.57369 | 0.000925 | -2.20197 | 0.015882 |
| TFPI | 1.700585 | 5.716612 | 3.562079 | 0.000957 | 3.250328 | 0.015882 |
| ECM2 | 1.951281 | 6.220575 | 3.526755 | 0.00106 | 3.867176 | 0.015882 |
| FOXM1 | 1.410511 | 7.744498 | -3.49561 | 0.001159 | 2.658314 | 0.015882 |
| PDPN | 1.99796 | 6.324915 | 3.493535 | 0.001166 | 3.994349 | 0.015882 |
| BICC1 | 2.126621 | 6.755107 | 3.482709 | 0.001203 | 4.366936 | 0.015882 |
| DCHS1 | 1.283424 | 7.140502 | 3.451484 | 0.001316 | 2.43416 | 0.015882 |
| MAP4K2 | -1.19892 | 5.409862 | -3.44977 | 0.001322 | -2.29568 | 0.015882 |
| FBXL7 | 1.311645 | 7.394482 | 3.432115 | 0.00139 | 2.482244 | 0.015882 |
| JAM3 | 1.264863 | 7.682606 | 3.426537 | 0.001413 | 2.403044 | 0.015882 |
| POLE2 | -1.10828 | 6.01334 | -3.4219 | 0.001431 | -2.15588 | 0.015882 |
| PLS3 | 1.070689 | 11.39187 | 3.416957 | 0.001452 | 2.100436 | 0.015882 |
| GEM | 1.853742 | 5.955311 | 3.25211 | 0.002309 | 3.614366 | 0.023858 |
| LHFPL6 | 1.263463 | 7.603347 | 3.20711 | 0.002616 | 2.400713 | 0.02525 |
| DACT1 | 1.526019 | 3.8142 | 3.174369 | 0.002863 | 2.879901 | 0.02525 |
| ZFP36 | 1.159976 | 9.974942 | 3.147939 | 0.003079 | 2.234536 | 0.02525 |
| NUAK1 | 1.146238 | 8.943608 | 3.119241 | 0.00333 | 2.21336 | 0.02525 |
| PSRC1 | -1.15782 | 8.015279 | -3.0935 | 0.003572 | -2.2312 | 0.02525 |
| MIS18BP1 | -1.07816 | 6.606418 | -3.08367 | 0.003669 | -2.11135 | 0.02525 |
| RAD54L | -1.15361 | 6.50937 | -3.07485 | 0.003758 | -2.22471 | 0.02525 |
| CLIP2 | 1.326334 | 6.685706 | 3.067972 | 0.003829 | 2.507646 | 0.02525 |
| LRRC17 | 1.44434 | 6.336302 | 3.066047 | 0.003849 | 2.721383 | 0.02525 |
| LMOD1 | 1.359963 | 5.034277 | 3.059526 | 0.003917 | 2.566787 | 0.02525 |
| NID2 | 1.721564 | 8.862003 | 3.048086 | 0.00404 | 3.297938 | 0.02525 |
| HJURP | -1.06285 | 7.933834 | -3.03712 | 0.004161 | -2.08905 | 0.02525 |
| PIMREG | -1.16897 | 6.688907 | -3.02232 | 0.004331 | -2.24851 | 0.02525 |
| CDC7 | -1.00892 | 8.028834 | -3.02116 | 0.004344 | -2.01241 | 0.02525 |
| LDB2 | 1.096171 | 7.0959 | 3.000146 | 0.004596 | 2.137865 | 0.025906 |
| FHL2 | 1.154055 | 10.12646 | 2.948715 | 0.005272 | 2.225385 | 0.02715 |
| KIT | 1.125145 | 4.351403 | 2.94574 | 0.005314 | 2.181235 | 0.02715 |
| PDZRN3 | 1.368351 | 9.336317 | 2.923579 | 0.005636 | 2.581752 | 0.02715 |
| AURKB | -1.13456 | 6.962698 | -2.92305 | 0.005643 | -2.19552 | 0.02715 |
| FLRT2 | -1.49155 | 7.839063 | 2.919708 | 0.005694 | -2.8119 | 0.02715 |
| TGFB1I1 | 1.176101 | 8.014173 | 2.907715 | 0.005877 | 2.259653 | 0.02715 |
| ABCA8 | 2.113317 | 5.243902 | 2.904782 | 0.005922 | 4.326848 | 0.02715 |
| PAQR4 | -1.15376 | 7.254754 | -2.88609 | 0.006221 | -2.22494 | 0.02715 |
| P3H2 | 1.641742 | 6.445609 | 2.88606 | 0.006222 | 3.120425 | 0.02715 |
| CDCA8 | -1.22406 | 7.082655 | -2.8799 | 0.006323 | -2.33603 | 0.02715 |
| CENPA | -1.07169 | 8.194276 | -2.87311 | 0.006437 | -2.10189 | 0.02715 |
| BAMBI | 1.66729 | 5.998542 | 2.86537 | 0.006569 | 3.176173 | 0.02715 |
| PLXDC1 | 1.106742 | 6.375125 | 2.851462 | 0.006812 | 2.153588 | 0.027544 |
| GPD2 | -1.10623 | 6.036807 | -2.83477 | 0.007115 | -2.15282 | 0.028158 |
| DUSP1 | 1.343239 | 11.50743 | 2.814423 | 0.007502 | 2.537203 | 0.028355 |
| OSR2 | 1.411669 | 7.166591 | 2.809057 | 0.007607 | 2.660447 | 0.028355 |
| RGS2 | 1.096386 | 8.905245 | 2.807224 | 0.007643 | 2.138183 | 0.028355 |
| SPINK5 | -1.68576 | 6.557589 | -2.80007 | 0.007786 | -3.2171 | 0.028355 |
| IGFBP4 | 1.52483 | 8.92931 | 2.787523 | 0.008043 | 2.877527 | 0.028355 |
| HTRA1 | 1.145164 | 10.46277 | 2.784957 | 0.008096 | 2.211713 | 0.028355 |
| OLFML1 | 1.233281 | 4.661019 | 2.776683 | 0.008271 | 2.35101 | 0.028355 |
| CORO2B | 1.502275 | 5.101271 | 2.771377 | 0.008384 | 2.83289 | 0.028355 |
| NR2F1 | 1.307398 | 8.724593 | 2.727676 | 0.009379 | 2.474947 | 0.03115 |
| CCN5 | 1.30084 | 2.892162 | 2.70548 | 0.009924 | 2.463724 | 0.032384 |
| CHST15 | 1.096118 | 9.57933 | 2.689503 | 0.010335 | 2.137787 | 0.033143 |
| SNAP91 | 1.018635 | 2.645548 | 2.681902 | 0.010536 | 2.026001 | 0.033194 |
| NDNF | 1.3546 | 4.075845 | 2.675501 | 0.010708 | 2.557261 | 0.033194 |
| EMILIN1 | 1.241812 | 6.880695 | 2.668303 | 0.010904 | 2.364954 | 0.033198 |
| ZFP69B | -1.14496 | 4.040907 | -2.66247 | 0.011066 | -2.2114 | 0.033198 |
| CDC20 | 1.029583 | 9.715055 | -2.65024 | 0.011412 | 2.041435 | 0.033692 |
| TXK | -1.29051 | 3.615809 | -2.63124 | 0.011969 | -2.44614 | 0.034698 |
| GFPT2 | 1.128325 | 4.967894 | 2.626064 | 0.012126 | 2.186047 | 0.034698 |
| FCGBP | 1.512422 | 7.911767 | 2.619804 | 0.012317 | 2.852887 | 0.034711 |
| CCN2 | 1.291488 | 10.1563 | 2.602626 | 0.012856 | 2.447804 | 0.035691 |
| NBL1 | 1.226406 | 8.97529 | 2.575831 | 0.013741 | 2.339834 | 0.037586 |
| GHR | 1.354641 | 4.232007 | 2.567713 | 0.01402 | 2.557334 | 0.037604 |
| IL12A | -1.22332 | 3.273814 | -2.55717 | 0.01439 | -2.33483 | 0.037604 |
| KLF2 | 1.067341 | 8.37353 | 2.554954 | 0.014469 | 2.095567 | 0.037604 |
| ANGPTL4 | 1.048256 | 5.368723 | 2.552499 | 0.014557 | 2.068029 | 0.037604 |
| NR4A1 | 1.322153 | 6.932428 | 2.512722 | 0.016049 | 2.500389 | 0.040893 |
| EGR2 | 1.178688 | 7.78475 | 2.506513 | 0.016295 | 2.263709 | 0.040957 |
| FAT1 | 1.000169 | 10.04318 | 2.497491 | 0.016657 | 2.000235 | 0.04131 |
| ACTA2 | 1.137667 | 11.18711 | 2.483609 | 0.017229 | 2.20025 | 0.041657 |
| PDGFRA | 1.567588 | 9.429396 | 2.483231 | 0.017245 | 2.964086 | 0.041657 |
| ASF1B | -1.06013 | 7.587243 | -2.4611 | 0.018195 | -2.08512 | 0.042503 |
| SYCP2 | -1.32573 | 5.055069 | -2.45997 | 0.018244 | -2.5066 | 0.042503 |
| FLG | 1.448872 | 2.972198 | 2.455855 | 0.018427 | 2.729945 | 0.042503 |
| H3C10 | -1.50607 | 5.234114 | -2.454 | 0.018509 | -2.84035 | 0.042503 |
| KIF18B | -1.01308 | 8.053595 | -2.38514 | 0.021827 | -2.01822 | 0.04951 |
